# The Multifunctional Host Defense Peptide SPLUNC1 Is Critical for Homeostasis of the Mammalian Upper Airway

**DOI:** 10.1371/journal.pone.0013224

**Published:** 2010-10-07

**Authors:** Glen McGillivary, Lauren O. Bakaletz

**Affiliations:** Center for Microbial Pathogenesis, The Research Institute at Nationwide Children's Hospital, The Ohio State University College of Medicine, Columbus, Ohio, United States of America; National Institute of Allergy and Infectious Diseases, National Institutes of Health, United States of America

## Abstract

Otitis media (OM) is a highly prevalent pediatric disease caused by normal flora of the nasopharynx that ascend the Eustachian tube and enter the middle ear. As OM is a disease of opportunity, it is critical to gain an increased understanding of immune system components that are operational in the upper airway and aid in prevention of this disease. SPLUNC1 is an antimicrobial host defense peptide that is hypothesized to contribute to the health of the airway both through bactericidal and non-bactericidal mechanisms. We used small interfering RNA (siRNA) technology to knock down expression of the chinchilla ortholog of human SPLUNC1 (cSPLUNC1) to begin to determine the role that this protein played in prevention of OM. We showed that knock down of cSPLUNC1 expression did not impact survival of nontypeable *Haemophilus influenzae*, a predominant causative agent of OM, in the chinchilla middle ear under the conditions tested. In contrast, expression of cSPLUNC1 was essential for maintenance of middle ear pressure and efficient mucociliary clearance, key defense mechanisms of the tubotympanum. Collectively, our data have provided the first *in vivo* evidence that cSPLUNC1 functions to maintain homeostasis of the upper airway and, thereby, is critical for protection of the middle ear.

## Introduction

Otitis media (OM), or inflammation of the middle ear, is the second most common pediatric infectious disease. Over 80% of children develop a least one incident of OM by three years of age and more than half of children experience multiple episodes of this disease [1], [2]. OM is the leading cause for physician office visits and pediatric surgeries and is the most frequent reason for the prescribing of antibiotics. Indeed, the estimated cost per episode of OM in the US is in the hundreds of dollars and more than 15 million antibiotic prescriptions are written each year for the treatment of OM. Further, the total cost of diagnosis and management of OM exceeds $5 billion annually in the US alone which serves to underscore the need to develop more effective preventative and therapeutic strategies for this prevalent disease [3], [4], [5], [6].

OM is not caused by highly virulent microorganisms, but is instead induced by a subset of commensal bacteria that comprise the normal flora of the pediatric nasopharynx. Three bacterial species (nontypeable *Haemophilus influenzae* [NTHI], *Streptococcus pneumoniae*, and *Moraxella catarrhalis*) are commonly isolated from middle ear effusions, with the greatest number of cases of OM with effusion caused by NTHI [7], [8]. When host airway defenses are compromised, most typically by upper respiratory tract (URT) viruses, these bacteria can behave as opportunistic pathogens and ascend the Eustachian tube (ET) to gain access to the middle ear [9], [10], [11]. As such, it is important to understand and characterize host defense mechanisms that contribute to prevention of OM and promote overall health of the uppermost respiratory tract.

In the mammalian airway, there are multiple first lines of defense that include mucociliary clearance, trapping functions of mucus glycoproteins and action of surfactants [12]. Several surfactant proteins are produced in the ET and these proteins serve to lower surface tension at the mucosal surface, an important parameter for optimal ET function [12]. Additionally, some of these surfactant proteins are members of the antimicrobial host defense family of proteins (APs) and protect mucosal surfaces by direct agglutination and opsonization of microorganisms [12]. The AP family of proteins, which also includes lysozyme, lactoferrin, cathelicidins, peptidoglycan recognition proteins, and β-defensins, are key components of the primary defense system and can inactivate bacteria, fungi and viruses on epithelial surfaces that line the respiratory tract [13], [14], [15], [16], [17]. We have previously demonstrated that the chinchilla, a predominant host used to model human OM, produces several APs in the upper airway including a β-defensin, and that expression of this defensin at the mucosal surface directly impacts the ability of NTHI to colonize the nasopharynx [18]. Collectively, these data provide evidence that effectors of innate immunity are produced at sites relevant to OM and suggest that these proteins likely play a critical role in defense of the middle ear.

The SPLUNC1 protein, a recently described AP, is a predominant constituent of surface liquid that covers mucosal surfaces of the human respiratory tract [19], [20]. SPLUNC1 is a secreted protein and has been detected in both nasopharyngeal lavage (NL) fluid [21] and sputum [22]. This AP is produced by mucosal tissues of the airway including the nasal septum, ethmoid turbinates, nasopharynx, trachea and lung [19], [22], [23], [24]. Specifically, SPLUNC1 is predominately produced by mucous cells and submucosal glands, although expression by epithelial cells of the upper airway has also been reported [19], [23]. With regards to SPLUNC1 function, purified recombinant protein can kill bacteria and also reduce biofilm formation by *Pseudomonas aeruginosa in vitro*, supporting the assertion that this molecule functions in host defense [19], [20], [25], [26], [27]. In addition, SPLUNC1 is a multifunctional protein that serves to protect the sodium channel protein ENaC, which regulates airway surface liquid volume, from proteolytic cleavage. Effective maintenance of mucosal liquid volume, mediated by appropriate expression of SPLUNC1, is thought to promote mucociliary clearance of microorganisms in the airway [20], a key host defense mechanism utilized in protection of the middle ear [9]. Additionally, human SPLUNC1 has been reported to act as a surfactant to reduce surface tension at an air-liquid interface [28], thus providing an additional mechanism by which this AP likely contributes to proper airway function. Altogether, data obtained from these *in vitro* analyses suggests that SPLUNC1 is an AP operational at mucosal surfaces that likely promotes health of the airway through bactericidal and non-bactericidal mechanisms.

As SPLUNC1 is postulated to play a role in host defense against microorganisms, we utilized small interfering RNA (siRNA) technology to knock down expression of the chinchilla ortholog of human SPLUNC1 (cSPLUNC1) and determined the impact of altered cSPLUNC1 expression on the development of experimental OM induced by NTHI. Under the conditions tested, chinchillas administered a cSPLUNC1-specific siRNA, then directly challenged with NTHI in the middle ear did not show a significant difference in the concentration of bacteria within the tympanum, compared to animals that received a control siRNA that did not modulate expression of cSPLUNC1. In contrast however, reduced cSPLUNC1 expression had a major impact on ET function as evidenced by a pronounced deficiency in the ability of this organ to maintain proper middle ear pressure or mediate effective mucociliary clearance, compared to controls. We thus provided evidence that ET dysfunction observed in animals with diminished cSPLUNC1 expression was likely due to the intrinsic ability of this AP to function as a biological surfactant. Collectively, our data suggested that the surfactant activity of cSPLUNC1 was essential for homeostasis of the uppermost airway and therefore, defense of the middle ear.

## Results

### Cloning of cSPLUNC1 cDNA

To begin to determine the role that SPLUNC1 might play in prevention of bacterial OM, we cloned a 792 bp cDNA that encoded the chinchilla ortholog of human SPLUNC1 (cSPLUNC1). The cSPLUNC1 cDNA was predicted to encode a 263 amino acid protein with an abundance of hydrophobic residues (117 amino acids, 44%) and a molecular mass of 27.3 kDa. CLUSTAL W analysis of the deduced cSPLUNC1 amino acid sequence demonstrated strong conservation with human (74.6% identity) and rat (68.6% identity) SPLUNC1 [supporting information (SI) Fig. S1]. In addition, bioinformatic tools predicted that cSPLUNC1, similar to human SPLUNC1, was a secreted protein (Fig. S1). Collectively, these data suggested that cSPLUNC1 likely shared activities with human SPLUNC1 such as the ability to kill bacteria [25], [26] and act as a surfactant *in vitro* [29].

### Determination of relative expression of cSPLUNC1 mRNA and protein in chinchilla tissues

**Figure 1:**
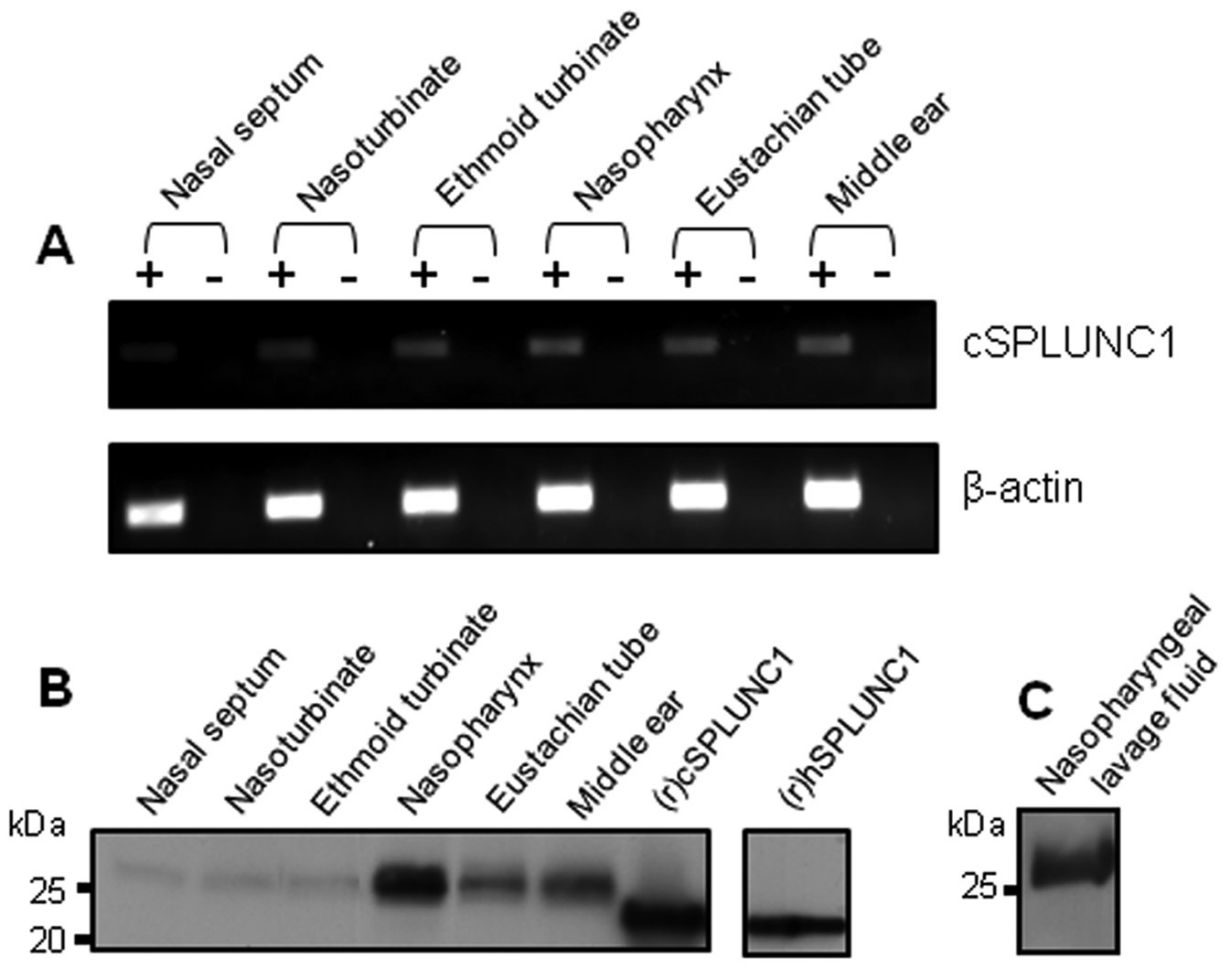
cSPLUNC1 mRNA and protein expression in the chinchilla upper airway. (A) RT-PCR analysis of (top panel) cSPLUNC1 or (bottom panel) β-actin transcripts in multiple chinchilla tissue homogenates. β-actin mRNA was amplified to confirm that equal amounts of template RNA was used in the amplification process. Amplicons generated with reverse transcriptase (+) or without reverse transcriptase (−) were separated in an ethidium bromide-stained agarose gel. (B) We used anti-human SPLUNC1 in Western blot to detect native cSPLUNC1 in selected mucosal homogenates. Recombinant cSPLUNC1 [(r)cSPLUNC1] was also detected to provide evidence that the antisera used in this analysis recognized the chinchilla ortholog of human SPLUNC1 and recombinant human SPLUNC1 [(r)hSPLUNC1] served as the positive control in this analysis. (C) Proteins in nasopharyngeal lavage fluid were separated by SDS-PAGE and Western blot was used to detect secreted cSPLUNC1 in the upper airway. cSPLUNC1 mRNA and protein was detected in every URT tissue evaluated.

To enable our ability to attempt to knock down expression of cSPLUNC1 *in vivo*, it was necessary to first identify tissues in the upper airway that expressed cSPLUNC1. We therefore isolated total RNA from several URT tissues and used RT-PCR to amplify cSPLUNC1 transcripts. cSPLUNC1 mRNA was detected in each URT sample evaluated which included mucosa from the nasal septum, nasoturbinate, ethmoid turbinate, nasopharynx, Eustachian tube, and middle ear (Fig. 1A). We also amplified β-actin from these same tissues as a control (Fig. 1A).

To complement our analysis of cSPLUNC1 mRNA expression, we used Western blot to detect cSPLUNC1 protein in the chinchilla upper airway. We reasoned that antibodies directed against human SPLUNC1 would also detect cSPLUNC1, and indeed demonstrated that this antiserum recognized recombinant cSPLUNC1 [(r)cSPLUNC1] (Fig. 1B). As a reference, we also detected recombinant human SPLUNC1 in the same immunoblot (Fig. 1B). We subsequently showed that native cSPLUNC1 was produced in mucosa of the nasal septum, nasoturbinate, ethmoid turbinate, nasopharynx, Eustachian tube, and middle ear (Fig. 1B), a finding that was in full agreement with results obtained from our RT-PCR analysis. In addition, we demonstrated that cSPLUNC1 was secreted in the airway by detection of this protein in chinchilla NL fluids (Fig. 1C). Interestingly, recombinant cSPLUNC1 separated by SDS-PAGE did not exhibit the same mobility as native cSPLUNC1. This observation was due to the absence of eukaryotic post-translation modifications such as glycosylation which has been reported for human SPLUNC1 present in NL fluid [30] and which we have obtained evidence for native cSPLUNC1 (McGillivary *et al*, unpublished). Collectively, our results demonstrated that cSPLUNC1 was produced at several mucosal sites in the chinchilla URT and suggested that these tissues were indeed appropriate targets for attempting to silence cSPLUNC1 expression.

### Administration and detection of siRNA in the chinchilla upper airway

**Figure 2:**
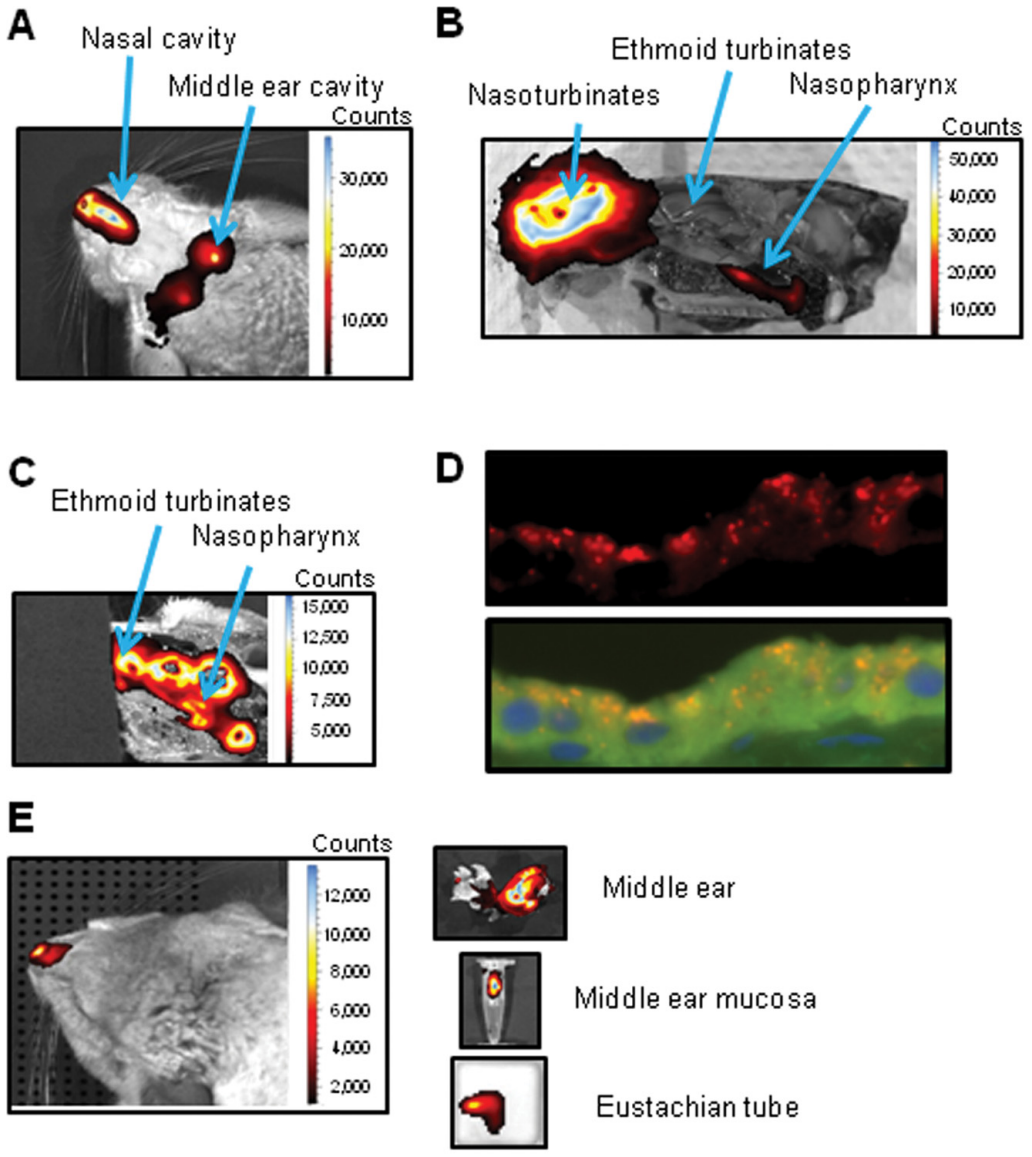
Detection of siRNA in the chinchilla upper airway. A negative control siRNA labeled with Alexafluor-647 was administered intranasally and transbullarly to chinchillas and fluorescence was detected (A) immediately, (B, C, and D) three hours, or (E) five days after delivery of siRNA. The images in (B and C) are of a sagittal section of a chinchilla head with tissues that express cSPLUNC1 identified by arrows. The nasoturbinates in (C) are covered by black non-fluorescent paper to block the strong fluorescence from this tissue and to allow detection of the weaker fluorescence from siRNA in the ethmoid turbinates and nasopharynx. (D) Nasoturbinate mucosae was embedded, sectioned, and stained with phalloidin conjugated to FITC to detect actin (green) and DAPI to stain DNA (blue). The top panel shows the fluorescently-labeled siRNA (red) alone and the bottom panel shows labeling for siRNA, actin, and DAPI. (E, right panel) *Ex vivo* analysis of the presence of siRNA in the tubotympanum. These data demonstrated that siRNA entered epithelial cells of the upper airway and showed our ability to detect siRNA in the chinchilla nasal cavity, Eustachian tube, and middle ear for at least five days after delivery of siRNA.

siRNA has not previously been used in the chinchilla host to generate a knock down in gene expression. As such, it was necessary for us to develop an siRNA approach to knock down expression of cSPLUNC1 *in vivo*. Chinchillas were intranasally and transbullarly administered 10 nmoles of a siRNA labeled with alexafluor-647 and fluorescence was subsequently monitored in the airway. We first used a non-silencing siRNA to minimize any impact that gene knock down might have on our ability to track the nucleic acid *in vivo*. We detected siRNA in the nasal cavity and middle ear of chinchillas immediately after delivery of the molecule to these sites (Fig. 2A), whereas no fluorescence was detected in animals that did not receive siRNA (data not shown). Three hours after administration of siRNA, we sacrificed a chinchilla to determine the precise location of siRNA within the nasal cavity. Fluorescence was readily observed in the nasoturbinates and as far retrograde as the nasopharynx, but was not noted in the ethmoid turbinates (Fig. 2B). The computer software used in this analysis scales output to the greatest signal measured within a single scan and therefore we reasoned that the observed very strong fluorescence in the nasoturbinates could have masked weaker fluorescence in the ethmoid turbinates. Therefore, we covered the nasoturbinates to block fluorescence from this tissue and were now able to show that siRNA was indeed delivered to the ethmoid turbinates (Fig. 2C). To provide evidence that siRNA not only reached sites of cSPLUNC1 expression but also entered epithelial cells where gene silencing is mediated, we demonstrated by fluorescence microscopy that delivery of siRNA to the chinchilla nasal cavity resulted in the presence of siRNA within the cytoplasm of epithelial cells of the upper airway (Fig. 2D).

To next determine the kinetics for detection of siRNA *in vivo*, animals were imaged one, two, four, and five days after administration of the molecule. We detected fluorescence in the nasal cavity of chinchillas for at least five days (Fig. 2E left panel) and for a minimum of two days in the middle ear (data not shown). Upon sacrifice of chinchillas five days after delivery of siRNA, we demonstrated by *ex vivo* imaging that siRNA was indeed present in the middle ear, in excised middle ear mucosa, and in ET tissues (Fig. 2E right panel). These results demonstrated that siRNA delivered to the nasal cavity and middle ear of chinchillas was maintained in these tissues for a minimum of five days and suggested that a knock down in cSPLUNC1 gene expression could likely be similarly maintained for this duration.

### Determination of cSPLUNC1 knock down *in vivo*

**Figure 3:**
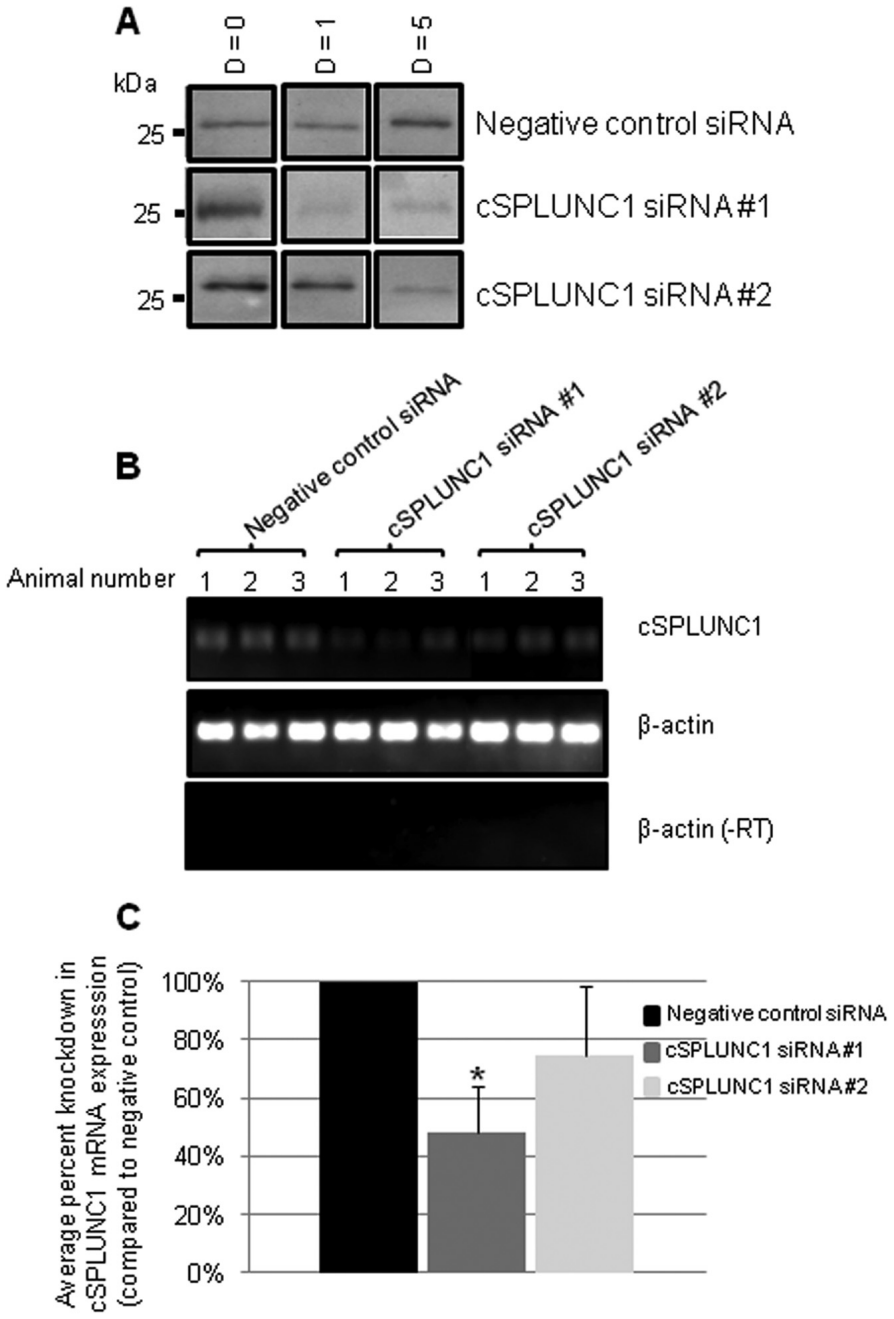
Knock down of cSPLUNC1 expression *in vivo*. (A) NL fluids were collected from chinchillas prior to administration of siRNA (d = 0) or after delivery of 10 nmoles of an siRNA. Two unique siRNAs that were designed based on the cDNA sequence of cSPLUNC1 and an siRNA that did not have complementary sequence to cSPLUNC1 were intranasally delivered to chinchillas, and NL fluids were collected from animals one and five days later. cSPLUNC1 protein present in NL fluids was detected by Western blot using rabbit anti-native cSPLUNC1 and knock down in expression was determined by comparison of band intensities for each panel before and after delivery of siRNA. (B) The siRNA molecules utilized in (A) were administered intranasally to chinchillas (n = 3 per cohort) and 24 hours later animals were sacrificed, nasoturbinate mucosae was collected, and total RNA was isolated. RT-PCR with primers designed to amplify (top panel) cSPLUNC1 or (middle and bottom panels) the control β-actin was used to determine relative mRNA expression for the targets among the cohorts. Amplification was also done in the absence of reverse transcriptase to demonstrate the absence of contaminating DNA as shown in the bottom panel. (C) Densitometric analysis of the intensity of RT-PCR amplicons shown in (B). Administration of 10 nmoles of cSPLUNC1 siRNA #1 resulted in a significant reduction (asterisk) in cSPLUNC1 expression, compared to the negative control siRNA.

To now demonstrate that we could indeed silence cSPLUNC1 expression in the airway, we intranasally administered to chinchillas one of several doses (0.05, 0.5, 5, or 10 nmoles) of two unique siRNAs that were designed to knock down expression of cSPLUNC1. We collected NL fluids from chinchillas before siRNA treatment to determine baseline cSPLUNC1 expression in these animals and used Western blot to determine protein expression over time. We did not observe a significant decrease in cSPLUNC1 expression when 0.05, 0.5, or 5 nmoles of siRNA (cSPLUNC1 siRNA #1 or #2) was administered to chinchillas (data not shown). In contrast, delivery of 10 nmoles of siRNA #1 to the nasal cavity resulted in diminished expression of cSPLUNC1 after one and five days after delivery of siRNA, when compared to cSPLUNC1 abundance from the same animal before administration of siRNA (Fig. 3A). Delivery of siRNA #2 resulted in a more modest and delayed reduction in cSPLUNC1 expression, while administration of 10 nmoles of negative control siRNA did not result in a detectable reduction in cSPLUNC1 expression at any time point evaluated (Fig. 3A).

To provide additional evidence for siRNA-mediated knock down in cSPLUNC1 expression, we analyzed cSPLUNC1 mRNA expression in nasoturbinate mucosae of the upper airway one day after delivery of siRNA to chinchillas (n = 3 animals per cohort). RT-PCR analysis demonstrated that administration of cSPLUNC1 siRNA #1 resulted in a 52% reduction (*p = *0.004), whereas delivery of cSPLUNC1 siRNA #2 resulted in a 25% reduction (*p>*0.05) in cSPLUNC1 mRNA expression, compared to animals that received the negative control siRNA (Fig. 3B and C). Collectively, these data provided the first demonstration of a gene knock down in the chinchilla.

### Purification of native cSPLUNC1 and determination of its ability to kill NTHI

**Figure 4:**
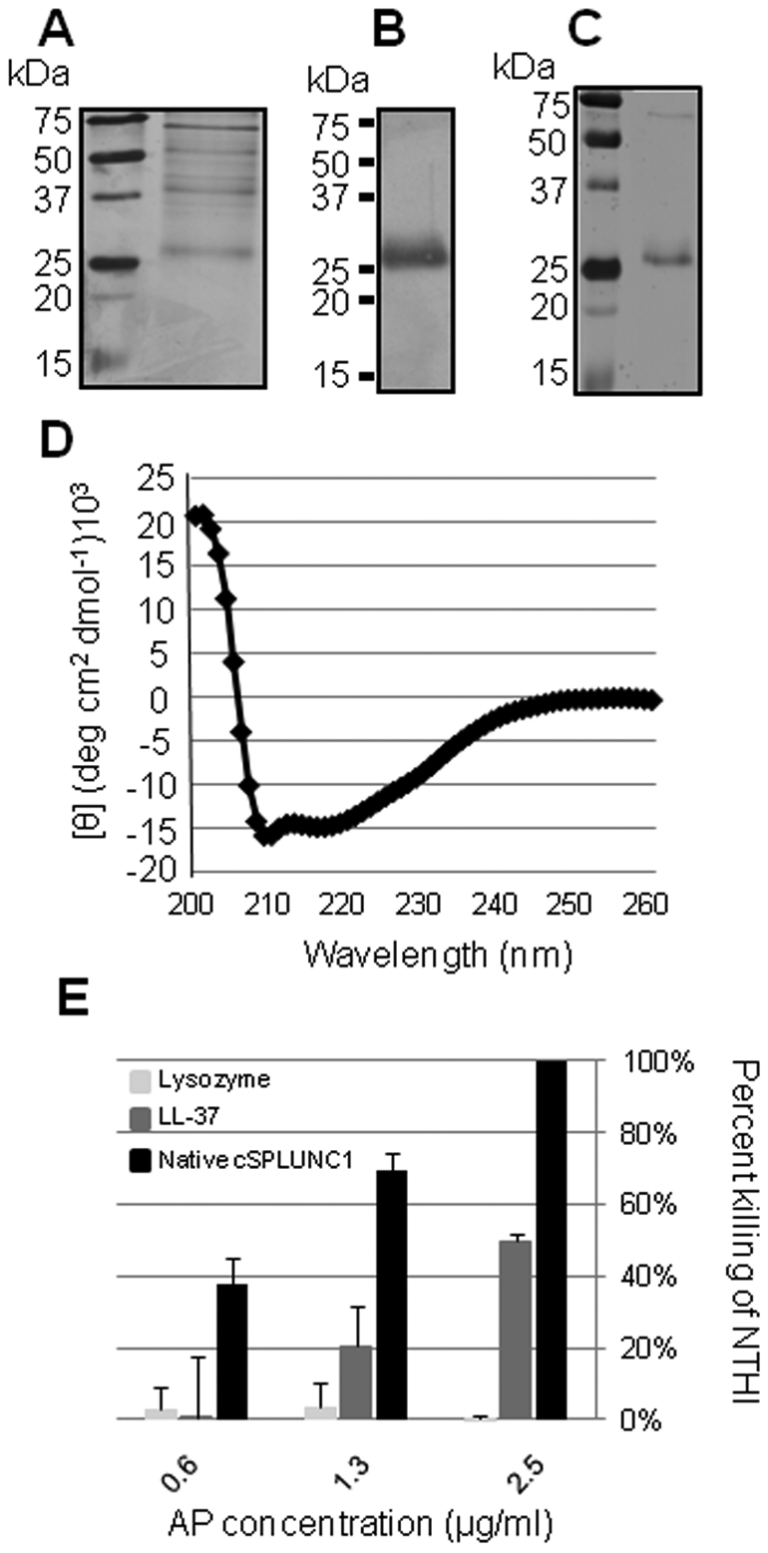
Purification of native cSPLUNC1 and its ability to kill NTHI. Secreted proteins obtained from washing the apical surface of CNPEs (Fig. S2) with PBS were separated by SDS-PAGE and (A) silver stained or (B) used in Western blot to show native cSPLUNC1. Additionally, proteins collected from the surface of CNPEs were separated by SDS-PAGE, and cSPLUNC1 was gel extracted, electroeluted, and refolded to obtain native protein. (C) Purified cSPLUNC1 was detected in a silver stained SDS-PAGE gel and (D) shown to exhibit secondary structure as determined by circular dichroism analysis. (E) NTHI 86-028NP was incubated with increasing concentrations of native cSPLUNC1, LL-37, or lysozyme for 1 hour, and the number of surviving colony forming units were determined. Native cSPLUNC1 demonstrated bactericidal activity against NTHI *in vitro* which suggested that cSPLUNC1 may also impact the ability of NTHI to survive *in vivo*.

As stated earlier, SPLUNC1 is a multifunctional protein that is able to kill bacteria [20], [25], [26], [27], [28], [30]. As NTHI is a predominant causative agent of OM, we first demonstrated that isolated native cSPLUNC1 was able to kill NTHI *in vitro* (Fig. 4A, B, C, D, and E). We showed that our purified protein reacted with SPLUNC1-specific antiserum in Western blot (Fig. 4B) and capillary-liquid chromatography-nanospray tandem mass spectrometry (LC/MS/MS) analysis provided sequence which matched 75 of the 263 amino acids predicted from the cSPLUNC1 cDNA sequence. Further, we used circular dichroism to show that our purified protein exhibited secondary structure similar to a published report for recombinant human SPLUNC1 [28] with 67% of the molecule predicted to be alpha-helical, 3% as a β-sheet, and 30% in a random coil conformation (Fig. 4D). Interestingly, our purification scheme, that included electroelution of ∼25 kDa native cSPLUNC1 from SDS-PAGE gels, also resulted in detection of a ∼65 kDa band by SDS-PAGE (Fig. 4C). This protein was also identified by LC/MS/MS (53 of 263 amino acids) as cSPLUNC1 which suggested that native cSPLUNC1 was capable of forming a multimer *in vitro*.

After we demonstrated our ability to purify native cSPLUNC1, we next assessed the bactericidal activity of native cSPLUNC1 against NTHI and showed that 1.3 µg native cSPLUNC1/ml phosphate buffer was able to kill greater than 50% of a 1×10^5^ inoculum of NTHI in a one hour assay (Fig. 4). Our data also demonstrated that native cSPLUNC1 was more effective in killing NTHI than either the human cathelicidin LL-37 or lysozyme (Fig. 4), two additional APs that are expressed in the upper airway [31], [32]. As it has been estimated that SPLUNC1 is expressed in airway secretions at concentrations of 10–250 µg protein/ml fluid [28], these data suggested that as typically produced in the normal upper airway, cSPLUNC1 was likely able to kill NTHI *in vivo*.

### Effect of cSPLUNC1 knock down on the development of bacterial OM

**Figure 5:**
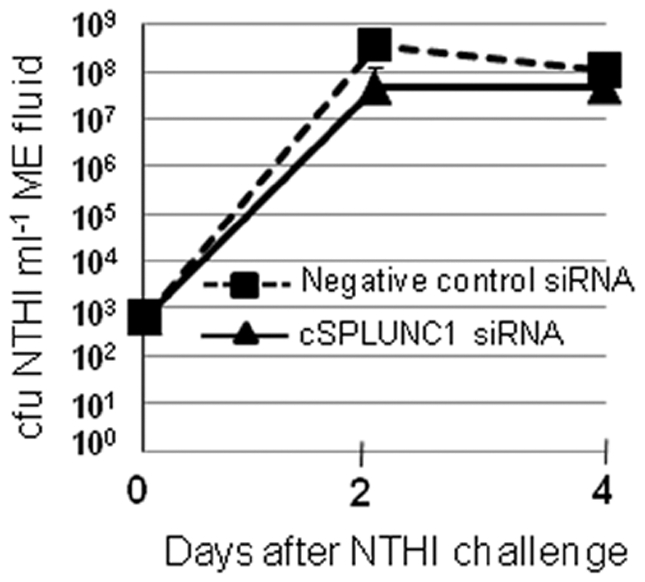
Effect of cSPLUNC1 knock down on the development of OM induced by NTHI. Chinchillas (3 per cohort) were administered either a control or cSPLUNC1 specific siRNA and challenged one day later with NTHI. The load of NTHI in middle ear effusions was subsequently determined two and four days after bacterial challenge. Knock down in cSPLUNC1 did not significantly impact survival of NTHI in the chinchilla middle ear under the conditions tested wherein a rigorous challenge dose was used.

We next determined the ability of cSPLUNC1 to impact NTHI survival in the chinchilla middle ear. Two cohorts of animals (n = 3 per cohort) were administered 10 nmoles of either a negative control siRNA or cSPLUNC1 siRNA #1. Twenty-four hours later, chinchillas were transbullarly challenged with NTHI and the resultant middle ear effusions were collected 2 and 4 days after bacterial challenge to determine the concentration of NTHI present in these middle ear fluids. We showed that at the challenge dose used (∼1000 cfu NTHI/ear), the load of NTHI present in effusions was not significantly different between animals that received either control or cSPLUNC1 siRNA wherein approximately 1×10^8^ cfu NTHI/ml middle ear fluid were present two days after bacterial challenge (Fig. 5). We also did not observe a significant difference between the two cohorts in the load of NTHI in the middle ears of animals four days after bacterial challenge (Fig. 5).

**Figure 6:**
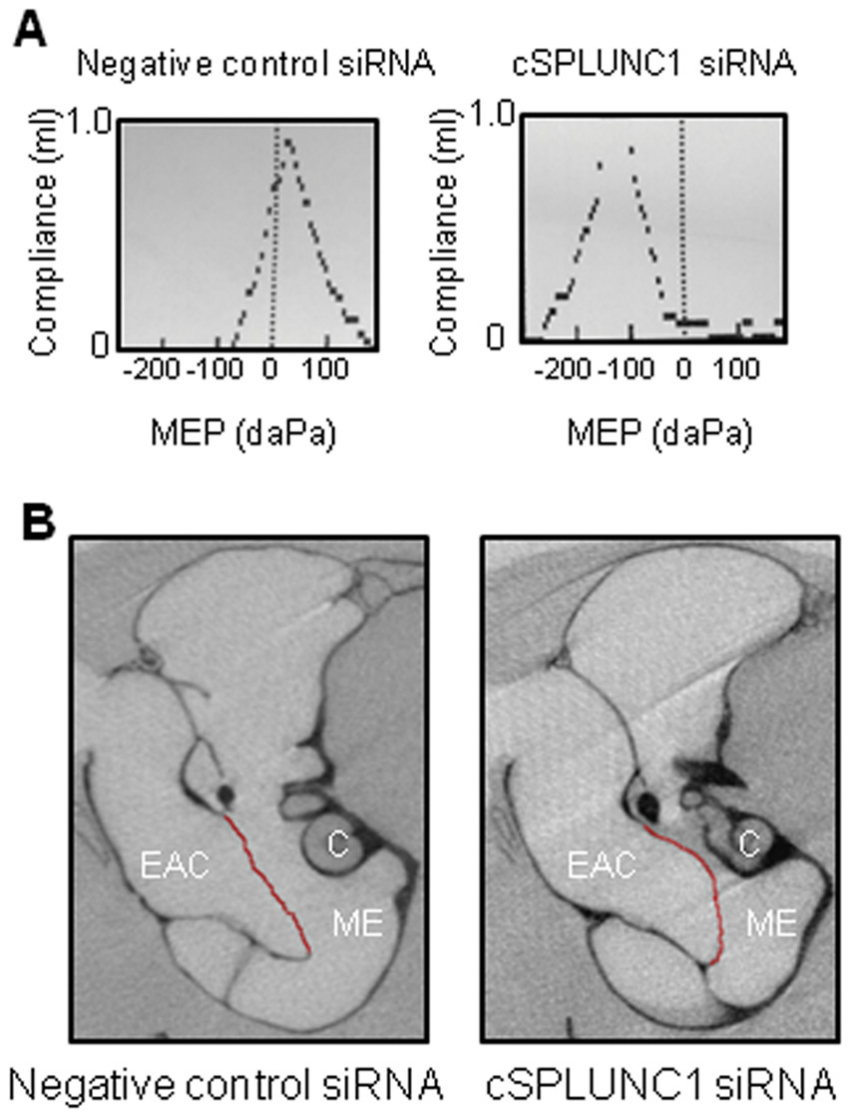
Impact of cSPLUNC1 knock down on the tubotympanum. Chinchillas were administered either a control or cSPLUNC1-specific siRNA and (A) tympanometry was used to determine middle ear pressure and tympanic membrane compliance and (B) computed tomography imaging was used to visualize the right middle ear cavity of chinchillas 24 hours after delivery of siRNA (before challenge with NTHI). The tympanic membrane in (B) was pseudo-colored red to highlight the retraction of the tympanic membrane that occurred in animals that received cSPLUNC1 siRNA. Other structural differences that are observed between the left and right panels (ie. morphology of the cochlea) are due to slight differences in the positioning of animals in the scanner and do not represent pathological changes in the ears of animals when expression of cSPLUNC1 was diminished. Images shown in (A) and (B) are representative of results obtained from the respective cohorts. Knock down of cSPLUNC1 resulted in a strong middle ear under pressure as evidenced by marked retraction of the tympanic membrane. EAC – external ear canal, ME- middle ear cavity, C- cochlea.

In addition to culture data, we used tympanometry to provide an indication of the relative severity of experimental OM in animals challenged with NTHI. We monitored changes in middle ear pressure (MEP) and tympanic membrane compliance (TMC), or mobility of the tympanic membrane, to obtain quantitative information on middle ear function. A tympanogram was considered abnormal if middle ear pressure was ≥−100 daPa or ≥+60 daPa and compliance values were ≤0.5 or ≥1.2 ml [33]. Our tympanometric analysis demonstrated that chinchillas administered cSPLUNC1-specific siRNA developed a strong negative MEP compared to controls. Chinchillas [n = 3 animals (6 ears) per cohort] that received control siRNA demonstrated both a normal average MEP value of 3±12 daPa and compliance value of 1±0.2 ml whereas chinchillas administered cSPLUNC1 siRNA exhibited an abnormally negative MEP of −117±12 daPa and associated increased compliance value of 1.5±0.2 ml. The difference in the average MEP and compliance values between the two cohorts was statistically significant (*p* = 0.0001 for MEP and *p* = 0.0001 for compliance). A representative image is shown in Fig. 6A. The pronounced shift to a negative MEP with a compliance value that was also abnormal, as assessed by the relative height of the curve in the tympanogram, suggested that the tympanic membrane was retracted and the mobility was thus altered (i.e. reduced) in animals administered cSPLUNC1-specific siRNA. We therefore directly visualized the middle ear cavity of chinchillas by micro-CT analysis and indeed observed that the tympanic membrane was retracted in animals that received cSPLUNC1 siRNA, a result not seen with those that received control siRNA (Fig. 6B). After challenge of chinchillas with NTHI, MEP measured in animals that received the cSPLUNC1 siRNA remained consistently more negative throughout the study compared to animals that received a control siRNA (data not shown). As a primary function of the ET is to equilibrate atmospheric pressure between the middle ear and the nasopharynx, these results suggested that silenced cSPLUNC1 expression in animals with NTHI-induced OM resulted in ET dysfunction.

### Determination of morphological changes in the tubotympanum after cSPLUNC1 knock down

**Figure 7:**
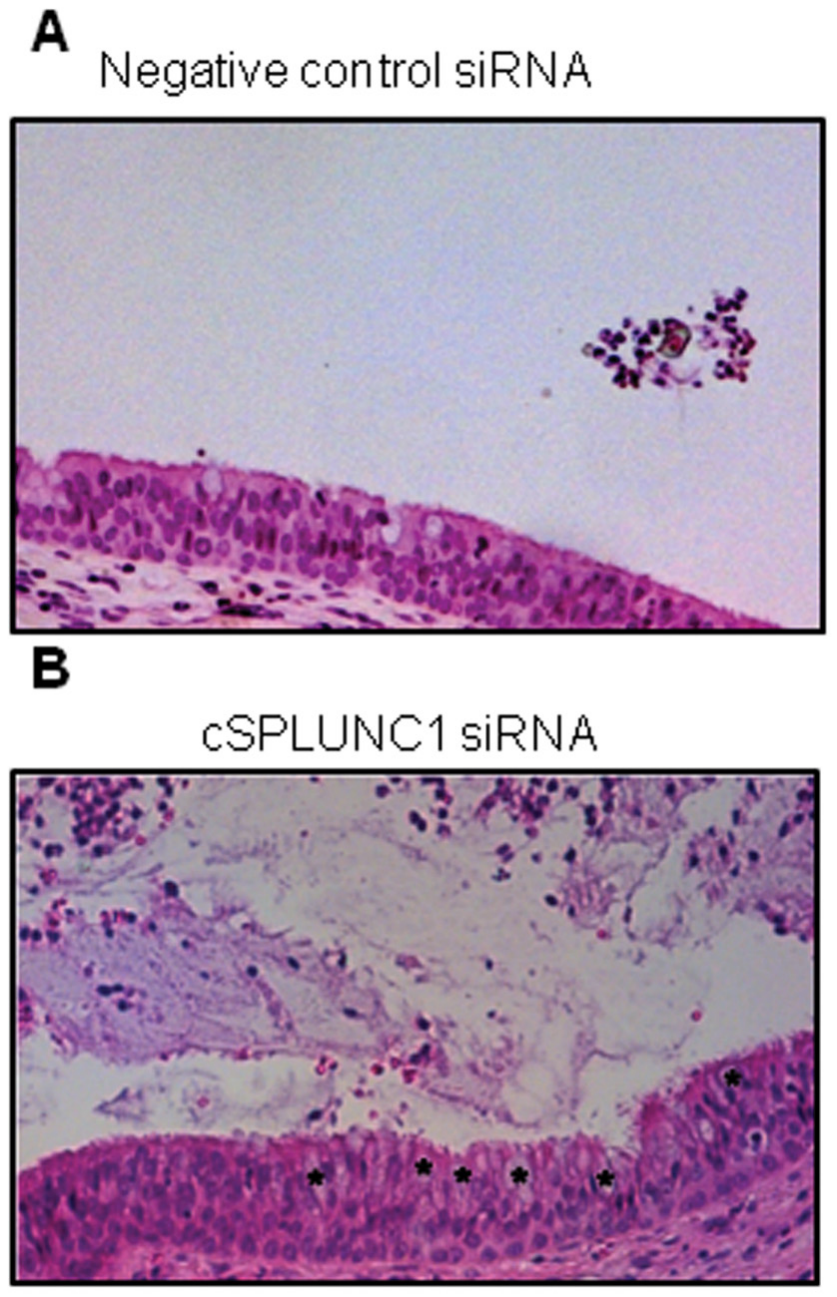
Effect of cSPLUNC1 knock down on the histopathology of the ET recovered from animals with NTHI-induced OM. Chinchillas (n = 2 per cohort) were administered either (A) a control or (B) cSPLUNC1-specific siRNA and challenged one day later with NTHI. After four days, animals were sacrificed and Eustachian tubes were embedded and stained with hematoxylin and eosin. Asterisks in (B) indicate goblet cells. Knock down in cSPLUNC1 expression in chinchillas with OM induced by NTHI resulted in a pronounced influx of inflammatory cells, accumulation of cellular debris and mucus, and goblet cell hyperplasia in the ET.

We next used a histological approach to begin to determine the underlying mechanism for the defect in ET function that we observed. Animals (n = 2 per cohort) that received control siRNA and were challenged with NTHI showed an inflammatory cellular infiltrate within the lumen of the ET but a predominately characteristic ciliated pseudo-stratified columnar epithelium with infrequent goblet cells evident (Fig. 7A). These results are consistent with what has been published previously when chinchillas are directly challenged in the middle ear with NTHI and an inflammatory host response is initiated [34]. In contrast, ETs from animals that received cSPLUNC1 siRNA demonstrated a more pronounced influx of immune cells, cellular debris and accumulation of mucus in the lumenal space (Fig. 7B). Goblet cell hyperplasia was also evident in the epithelium of the ET, and the increased number of these cells likely contributed to the increased presence of mucus in the ET lumen (Fig. 7B, asterisks). Decreased surfactant activity in the airway can result in increased production of mucins [35], and therefore our data suggested that the ET dysfunction we observed in chinchillas with NTHI-induced OM subsequent to silenced cSPLUNC1 expression was due to decreased surfactant activity in this tubal organ.

### Surfactant activity of native cSPLUNC1

**Figure 8:**
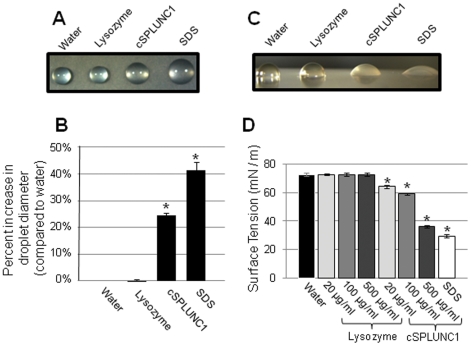
Surfactant activity of native cSPLUNC1. The relative ability of a droplet of water, lysozyme, native cSPLUNC1 or SDS to exhibit surfactant activity was determined via (A, B, and C) contact angle or (D) pendant drop tensiometry analysis. SDS was used as a positive control for surfactant activity whereas water and lysozyme served as the negative controls. (A, B, and C) Droplets of the four solutions were incubated on a hydrophobic surface and images from a (A) top or (C) side view were collected. (B) The diameter of each droplet was measured in millimeters (n = 4) and compared, as a percentage, to the water control which was set to a value of zero. (D) Surface tension of the four solutions was determined (n = 5) and the mean ± standard deviation presented. Asterisks denote a statistically significant difference (*p*<0.05) in values between samples and the water alone control. Similar to SDS, native cSPLUNC1 reduced surface tension of water and suggested that this AP acted as a surfactant *in vivo*.

To provide evidence that cSPLUNC1 indeed exhibited surfactant activity, we evaluated droplet contact angle to determine the relative ability of native cSPLUNC1 to reduce surface tension of water *in vitro*. In this approach, droplets of a solution were incubated on a hydrophobic surface and the ability of a protein to reduce surface tension was assessed by measuring the diameter of a droplet. We determined that 100 µg native cSPLUNC1/ml water demonstrated a 25% increase in the droplet diameter compared to water alone (*p* = 0.0003) (Fig. 8A and B). SDS, a detergent, also exhibited surfactant activity and served as a positive control (*p*<0.0001), while the negative control (lysozyme) did not show a significantly increased droplet diameter, relative to water (p>0.05). The greater ability of native cSPLUNC1 to act as a surfactant, compared to water or lysozyme, was also observed by reduced height of a native cSPLUNC1 droplet (Fig. 8C).

For a more quantitative analysis of cSPLUNC1 surfactant activity, we used the pendant drop method to directly measure surface tension. Droplets of water or lysozyme (20 µg/ml, 100 µg/ml, or 500 µg/ml) exhibited a surface tension of approximately 72 mN/m (Fig. 8D), results that were consistent with what is known from the literature [36]. In contrast, we showed that native cSPLUNC1 exhibited a dose dependent reduction in droplet surface tension wherein 20 µg cSPLUNC/ml water, the least concentrated sample tested, resulted in a statistically significant difference in surface tension (64 mN/m, *p*<0.0001), compared to water alone (Fig. 8D). The positive control (SDS) demonstrated a surface tension value of 29 mN/m (*p*<0.0001), a value that was similar to the surface tension of a droplet of native cSPLUNC1 when tested at a concentration of 500 µg/ml (Fig. 8D) (*p*<0.0001). These results showed that native cSPLUNC1, at estimated physiological concentrations [28], could indeed act as a surfactant and further supported the conclusion that, in the context of NTHI-induced OM, expression of cSPLUNC1 and its associated surfactant activity was crucial for proper ET function.

### Impact of silencing cSPLUNC1 expression on mucociliary clearance in the ET

**Figure 9:**
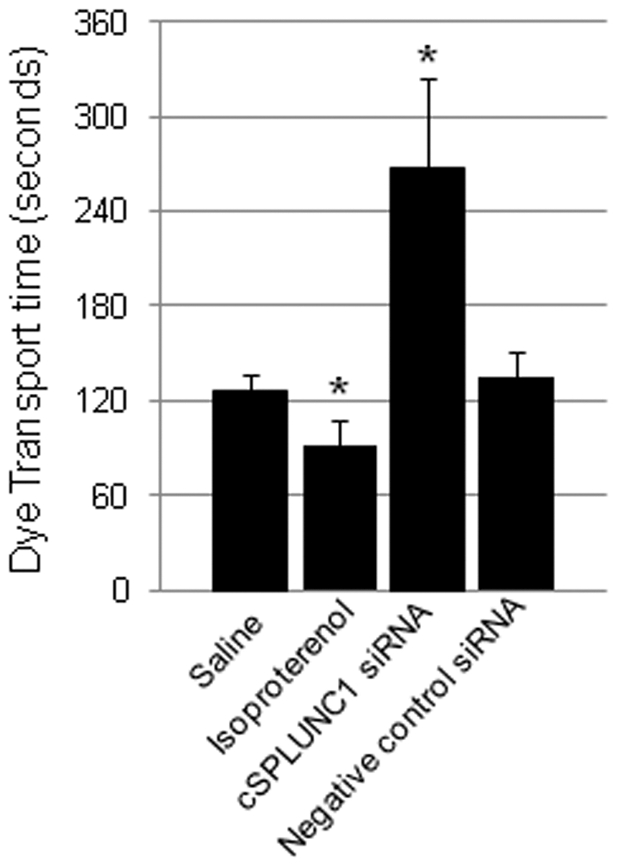
Role of cSPLUNC1 in ET function. Chinchillas (4 ears per cohort) were administered either saline, isoproterenol, cSPLUNC1 siRNA, or a negative control siRNA followed by delivery of a very small volume of dye into the middle ear cavity 24 hours later. The average transport time ± standard deviation (in seconds) required for dye to be transported from the middle ear to the nasopharyngeal orifice of the ET was determined for each cohort. Asterisks denote a statistically significant (*p*≤0.05) difference in dye transport time between the cohort that received saline alone and the cohorts that received isoproterenol or the cSPLUNC1 siRNA. Whereas the β-adrenergic mucociliary stimulator isoproterenol increased transport time, knock down in cSPLUNC1 expression conversely prolonged dye transport time likely as a result of reduced surfactant activity which diminished ET mucociliary clearance.

One biological function of the ET is to prevent entry of fluid and/or debris into the middle ear to promote proper functioning of the tympanum. As mucociliary clearance is impacted by expression of surfactant proteins [12], we next assessed the effectiveness of the mucociliary system of the ET in chinchillas that were administered either a negative control or cSPLUNC1-specific siRNA. Twenty-four hours after delivery of siRNA, we measured time required for the ET of chinchillas (4 ears per cohort) to transport a small bolus of Coomassie brilliant blue from the inferior aspect of the middle ear to the nasopharyngeal orifice of the ET (Fig. 9A). For animals that received saline, the average dye transport time was 126±10 seconds, whereas chinchillas that received the mucociliary stimulator isoproterenol exhibited a decreased transport time of 91±16 seconds (*p* = 0.009) (Fig. 9B). This latter result was expected as isoproterenol is a β-adrenergic stimulator known to increase ciliary beat frequency and thereby promote mucociliary clearance in the ET [37]. Animals that received negative control siRNA demonstrated a dye transport time of 134±17 seconds (*p*>0.05), similar to that of animals that received saline alone (Fig. 9B). In contrast, delivery of cSPLUNC1 siRNA resulted in a statistically significant increase in time, 268±56 seconds, required to translocate dye from the middle ear to the nasopharynx (*p* = 0.002). Thus, knock down of cSPLUNC1 expression resulted in an approximate 50% decrease in the ability of the ET to transport dye, compared to animals that received saline alone. Collectively, these results demonstrated that silenced expression of cSPLUNC1, and therefore also the associated reduced surfactant activity, diminished mucociliary clearance by the ET.

## Discussion

OM is a disease of opportunity wherein commensal bacteria present in the pediatric nasopharynx ascend the ET and enter the middle ear cavity, most typically after or concurrent with infection by URT viruses [7]. As such, characterization of host mechanisms that promote health of the uppermost airway provide important insight into the pathogenesis of OM and also help elucidate host factors that contribute to the prevention of this highly prevalent disease. In the mammalian airway, effectors of innate immunity such as APs defend mucosal surfaces and act to protect a host through bactericidal and non-bactericidal mechanisms [14], [15]. SPLUNC1 is an AP that is abundantly expressed in the upper airway [28], however the role that this protein might play in prevention of OM is not completely understood. Here, we utilized a siRNA-based approach to knock down expression of the chinchilla homologue of human SPLUNC1 and thereby demonstrated that this AP was critical for homeostasis of the tubotympanum.

As a first step in the knock down of cSPLUNC1 expression, we initially determined if we could deliver siRNA to tissues that expressed cSPLUNC1 and also assessed the length of time the molecule remained resident within the airway. We administered a fluorescently-labeled siRNA to the chinchilla URT and determined that we could detect this siRNA in the middle ear and nasal cavity. Specifically, intranasal administration of siRNA to the chinchilla airway allowed for detection of the molecule in the nasoturbinates, ethmoid turbinates and nasopharynx- sites known to express cSPLUNC1. After intranasal and transbullar administration of siRNA, we were able to detect fluorescence in the chinchilla nasal cavity for at least five days and could also observe fluorescence *ex vivo* in a dissected middle ear, excised middle ear mucosa, and ET tissues. Our results demonstrated that siRNA remained resident in the nasal cavity and middle ear for several days and suggested that we could similarly likely maintain a cSPLUNC1 knock down for this length of time *in vivo*. Indeed, we demonstrated that administration of cSPLUNC1-specific siRNA to the chinchilla airway resulted in a pronounced reduction in cSPLUNC1 mRNA and protein expression after one day and showed that protein expression was diminished for at least five days. It will be interesting to determine the total length of time that siRNA could be maintained in the nasal cavity, middle ear, and ET, as it has been reported that knock down of a target can last days to weeks depending on the dosing regimen [38].

Our data have provided the first demonstration of a successful gene knock down in the chinchilla host, and have further allowed us the opportunity to begin to assess the role that cSPLUNC1 played in experimental OM induced by NTHI. Although purified native cSPLUNC1 killed NTHI *in vitro*, the concentration of NTHI in middle ear fluids after transbullar challenge was not increased by a knock down in cSPLUNC1 expression, compared to controls. We speculate that the discrepancy between the *in vitro* and *in vivo* bactericidal activity of cSPLUNC1was likely due to the substantial growth of NTHI in middle ears following the robust challenge dose used here (ie. ∼1000 cfu NTHI/ear), where the bacterial load typically reaches ∼1×10^8^ cfu NTHI per ml of middle ear fluid within 48 hours. We are currently determining the role that cSPLUNC1 has on the ability of NTHI to colonize the chinchilla nasal cavity as the load of NTHI in this compartment (∼1×10^4^ cfu NTHI per ml NL fluid) is substantially less than that present within the middle ear after direct, transbullar challenge [18].

Interestingly, knock down in expression of cSPLUNC1 resulted in marked morphological and functional changes in the ET. Animals that were administered cSPLUNC1-specific siRNA showed a greater influx of immune cells into the ET lumen, compared to controls. Human SPLUNC-1 binds bacterial lipopolysaccharide which is thought to dampen the pro-inflammatory response [30], and this activity could explain the increased abundance of white cells in the ET. However, we could not detect a significant difference in expression of the pro-inflammatory cytokines IL-1, IL-6, IL-8, IFN- γ, or TNF-α in middle ear effusions from animals that received either control or cSPLUNC1-specific siRNA (data not shown), and therefore the underlying mechanism responsible for the increased presence of leukocytes in the ET remains to be described.

Silencing cSPLUNC1 expression in chinchillas with NTHI-induced OM also resulted in an inability of animals to properly equilibrate pressure between the middle ear and nasopharynx which contributed to retraction of the tympanic membrane, and also showed a greater accumulation of mucus in the ET lumen, compared to controls. It is known that diminished surfactant activity in the ET decreases the ability of this tubal organ to open effectively which results in impaired pressure regulation in the middle ear [12]. Also, it has been reported that surfactant protein C-deficient mice challenged with *Pseudomonas aeruginosa* augment mucin expression in the lung, compared to wild-type animals [35]. As we have demonstrated that cSPLUNC1 significantly affected surface tension *in vitro*, our data collectively suggested that cSPLUNC1 acted as a surfactant *in vivo* and that this activity of cSPLUNC1 was critical for proper ET function. We suggest that decreased surfactant activity in ETs from animals that received cSPLUNC1 siRNA also contributed to the diminished effectiveness of the ET mucociliary system since surfactants are known to have a major impact on this function [12]. We demonstrated that silencing cSPLUNC1 expression resulted in a 50% increase in the time required for the ET to transport a small bolus of dye from the middle ear to the nasopharynx. As mucociliary clearance is affected by rheological properties of the mucus blanket [9], it is likely that the increased mucus production in the ET of animals that received cSPLUNC1 siRNA contributed to the results that we observed. In addition, diminished cSPLUNC1 activity and therefore reduced surface liquid volume, in an ENaC-dependent manner, would likely result in altered airway hydration which could also impact mucociliary clearance [20].

A question remaining is why silencing cSPLUNC1 expression resulted in such a pronounced altered biological function when additional surfactant proteins are also produced in the Eustachian tube [12], [39], [40]. We suggest that diminished expression of cSPLUNC1 with its unique combination of activities that include binding of pro-inflammatory bacterial molecules, lowering of surface tension, and acting as a liquid volume sensor in the airway resulted in the establishment of an environment that could not be compensated for by the presence of additional surfactants, at least during the time frame that we evaluated. In a similar manner, investigators that utilize diverse animal models have also reported that altered expression of even a single effector of innate immunity can impact a disease course [41], [42]. As cSPLUNC1 played such an important role in maintaining normal function of the ET, we anticipate that cSPLUNC1 also contributes to the ability of the ET to prevent microbes such as NTHI from entering the middle ear as these microorganisms do not typically cause OM without prior compromise of immune defense [9]. If this hypothesis is indeed proven to be correct, then cSPLUNC1 expression would affect all three known physiologic functions of the ET that include pressure regulation, prevention of mucus and debris entry into the middle ear, and defense of the tympanum from microbes [16].

Collectively, we have provided the first evidence that the AP cSPLUNC1 is critical for proper ET function and serves to maintain homeostasis of the middle ear. In addition, these data also suggest that cSPLUNC1 contributes to normal protection of the middle ear and thereby, plays a key role in prevention of OM.

## Materials and Methods

### Ethics Statement

All studies that involved chinchillas were performed under protocol 01304AR, approved by The Research Institute at Nationwide Children's Hospital Institutional Animal Care and Use (IACUC) Committee.

### Animals

Healthy adult chinchillas (*Chinchilla lanigera*), were purchased from Rauscher's Chinchilla Ranch (LaRue, OH) and given chinchilla chow (Cincinnati Lab and Pet Supply, Cincinnati, OH) and water *ad libitum*. We collected NL fluids from animals that were anesthetized with xylazine (2 mg/kg, Fort Dodge Animal Health, Fort Dodge, IA) and ketamine (10 mg/kg, Phoenix Scientific Inc., St. Joseph, MO).

### Cloning of cSPLUNC1 cDNA

Human SPLUNC1 orthologs in the mouse, rat, and cow share significant pair-wise amino acid identities [43], which suggested that we could used PCR to amplify cSPLUNC1 cDNA. The CloneMiner system (Invitrogen, Carlsbad, CA) was used to construct a chinchilla cDNA library derived from pooled total RNA isolated from tissues of the URT (nasal septum, nasoturbinate, ethmoid turbinate, and nasopharynx). Total RNA was isolated from individual mucosal samples as reported previously [39] and suspended in a volume of 80 µl diethly pyrocarbonate-water. Fifty microliters from each of four RNA samples were combined into one tube, and the micropoly(A) purist system (Ambion, Austin, TX) was used to enrich for mRNA. Total RNA (211 µg), in a volume of 250 µl, was added to 250 µl of 2 X Binding Solution and the mixture was incubated with oligo(dT) cellulose. Cellulose with bound mRNA was pelleted, washed twice with Wash Solution 1 and 2, and RNA was eluted with 100 µl of RNA Storage Solution. An Agilent 2100 Bioanalyzer (Agilent, Foster City, CA) was used to confirm the integrity of purified RNA before and after mRNA enrichment. First strand cDNA was generated from a reaction that contained 2.5 µg mRNA, a CloneMiner biotin-*att*B2-Oligo(dT) primer (online supplement Table S1), and Superscript II reverse transcriptase (Invitrogen). *E. coli* DNA Ligase, DNA polymerase I, and RNase H were added to samples at 16°C to produce second strand cDNA. An *att*B1 adapter (Table S1) was ligated to the 5-prime end of the double-stranded cDNA, and the Qiaquick PCR purification system (Qiagen, Valencia, CA) removed residual un-ligated adapters and small (<100 bp) cDNA molecules.

The resultant cDNA was used in a Hotstart *Pfu* (Stratagene, La Jolla, CA) PCR reaction with the primers human SPLUNC1-F Internal (Table S1) and human SPLUNC1-R Internal (Table S1) used to amplify a partial cSPLUNC1 cDNA (∼300 bp). Amplicons were cloned into pCR-BLUNT II topo (Invitrogen) and transformed into *E. coli* Top10. We used M13 forward and reverse primers to sequence plasmids from selected recombinants that produced colonies on LB agar that contained 50 µg kanamycin/ml. One clone with sequence homology to SPLUNC1 genes from other mammals was evaluated further. To obtain a larger cSPLUNC1 cDNA clone, human SPLUNC1-F upstream (Table S1) and chinchilla SPLUNC1-R internal (Table S1) primers were utilized in a Hotstart *Pfu* PCR reaction with the chinchilla cDNA library again used as template. An ∼800 bp fragment was cloned into pCR-BLUNT II topo and sequenced as above. This plasmid (pGMSH-9) contained a DNA insert with significant similarity to human SPLUNC1 but did not encode the entire cSPLUNC1 open reading frame. Therefore, oligonucleotides were designed from the 5-prime untranslated region of cSPLUNC1 and the 3-prime untranslated sequence of the human SPLUNC1 cDNA (chinchilla SPLUNC1-F upstream and human SPLUNC1-R downstream [Table S1]). The complete 792-bp cSPLUNC1 cDNA was PCR amplified and cloned in pCR-BLUNT II topo to yield pGMSH-10. cSPLUNC1 cDNA sequence was submitted to the GenBank database (http://www.ncbi.nlm.nih.gov/Genbank/index.html) under accession number FJ830605.

Computer algorithms were used to further analyze the deduced amino acid sequence of cSPLUNC1. SignalP was used to identify the signal peptide cleavage site and a DNASTAR CLUSTAL W protein alignment was used to detect conserved regions between chinchilla, human, and rat SPLUNC1 molecules.

### Isolation of total RNA from chinchilla tissues and performance of RT-PCR

Total RNA was prepared from frozen tissues as described [18]. For RT-PCR, chinchilla SPLUNC1-F (Table S1) and chinchilla SPLUNC1-R (Table S1) at 0.5 µM each, with 2 nanograms of total RNA, were used in 25 µl total volume amplification reactions. As a control for loading of equal amounts of total RNA in this analysis, RT-PCR reactions that contained primers [chinchilla β-actin-F and chinchilla β-actin-R (Table S1)] to amplify chinchilla β-actin (Genbank accession number DQ826531) were also used. Amplicons generated from reactions with or without reverse transcriptase, to confirm absence of genomic DNA in RNA preparations, were separated in ethidium bromide-stained 0.8% agarose gels.

### Detection of cSPLUNC1 protein in the upper airway

Chinchilla tissues were homogenized in 0.5 ml sterile saline with a 1 X solution of protease inhibitors (Roche, Indianapolis, IN). Samples were centrifuged at 16,000×g for 1 minute and supernatants were removed. We determined the protein concentration of samples and equal amounts (30 µg) of protein were separated by SDS-PAGE. Proteins were transferred to nitrocellulose and blots were incubated with a 1∶1,000 dilution of mouse anti-human SPLUNC1 (R and D systems, Minneapolis, MN) followed by a 1∶10,000 dilution of a goat anti-mouse secondary antibody conjugated to horseradish peroxidase. The chemiluminescent ECL substrate (Amersham, Piscataway, NJ) allowed capture of signal on film.

### Ability to knock down expression of cSPLUNC1 *in vivo*

As an initial step towards the goal of generating a cSPLUNC1 knock down in the chinchilla upper airway, we first determined if we could deliver siRNA to tissues that expressed cSPLUNC1. We intranasally administered 10 nmoles of an Alexafluor 647-labeled negative control siRNA (Qiagen, Valencia, CA) in a 200 µl volume to animals via microaerosol sprayer (Wolfe Tory Medical, Salt Lake City, UT). siRNA was also delivered transbullarly to middle ears of chinchillas in a total volume of 200 µl saline. The Xenogen IVIS Spectrum system (Caliper life sciences, Hopkinton, MA) was used to detect the presence of fluorescently labeled siRNA in the upper airway of the chinchilla immediately, 3 hours, or 5 days after administration of siRNA.

To provide evidence that siRNA entered epithelial cells of the upper airway, one chinchilla that was administered fluorescently labeled siRNA was sacrificed 3 hours later. Mucosa from the nasoturbinates was dissected, frozen in O.C.T compound (Fisher Scientific, Pittsburgh, PA), and sectioned. Slides were fixed with cold acetone and non-specific binding was blocked with image-iT FX signal enhancer (Invitrogen). A 200 nM solution of FITC-conjugated phalloidin was used to label actin and DAPI (4′,6′-diamino-2-pheynylindole) was used to label DNA. Sections were viewed using a Zeiss Axiovert 200M microscope with apotome, and images were captured with an Axiocam MRm camera.

After establishment of an experimental approach to deliver siRNA to the upper airway, we intranasally administered either 0.05, 0.5, 5, or 10 nmoles of negative control siRNA or one of two siRNAs (cSPLUNC1 siRNA #1 and #2) designed to knock down expression of cSPLUNC1. The sequence for the sense strand of cSPLUNC1 siRNA #1 was GAAUUGAAAU UGCCCAUGtt and the sequence for cSPLUNC1 siRNA #2 was GAAUAACAUCAUCGAC UUAtt (Applied Biosystems, Foster City, CA). We obtained NL fluids as previously described [44] one and five days after delivery of siRNA. To detect cSPLUNC1, equal amounts of protein (30 µg) in NL fluids were separated by SDS-PAGE and Western blot was performed with a 1∶500 dilution of rabbit anti-native cSPLUNC1 as primary antibody (supporting information (SI) Materials and Methods S1). Blots were then incubated with a 1∶10,000 dilution of a goat anti-rabbit secondary antibody conjugated to horseradish peroxidase. The chemiluminescent ECL substrate (Amersham, Piscataway, NJ) allowed capture of signal on film. We could not provide a load control for this analysis as an antibody that recognized an appropriate chinchilla protein secreted in the airway was not readily available.

After determination of the optimal dose of siRNA required to reduce cSPLUNC1 protein production, we next confirmed the ability (or lack thereof) of these same siRNAs to diminish cSPLUNC1 mRNA expression *in vivo*. We intranasally administered 10 nmoles of cSPLUNC1 siRNA #1, cSPLUNC1 siRNA #2, or negative control siRNA to chinchillas (n = 3 per cohort) and sacrificed animals 24 hours later. Nasoturbinate mucosae was dissected, frozen in liquid nitrogen, and total RNA was prepared as described [18]. RT-PCR was performed as described above and a GS-800 calibrated densitometer (Bio-Rad, Hercules, CA) was used to determine pixel intensity of amplicons generated by RT-PCR. Mean values (± standard deviation) of the densitometric analysis were reported as the ratio of the pixel intensity of samples from chinchillas that received either cSPLUNC1 siRNA #1 or #2 compared to animals that received negative control siRNA. Statistical significance was calculated using a Student's *T*-test and was significance was accepted at a *p*-value of ≤0.05.

### Purification of native cSPLUNC1

To provide an abundant source of native cSPLUNC1, we first cultured primary cells derived from the chinchilla nasopharynx as this tissue was shown to produce native cSPLUNC1. Chinchilla nasopharyngeal epithelial cells (CNPEs) were allowed to polarize (Materials and Methods S1) and the apical surface of cells were washed every 2 days with 0.5 ml of PBS per well to recover secreted cSPLUNC1. Proteins collected from the apical surface of CNPEs were pooled and 5 ml of this sample was lyophilized overnight, suspended in 300 µl 1 X SDS-PAGE buffer without β-mercaptoethanol, and separated in a 15% SDS-PAGE gel. Native cSPLUNC1 was excised and electroeluted from the gel slice for 1½ hours at 100 Volts in a 3.5 kDa D-tube dialyzer (EMD, San Diego, CA). Samples were dialyzed overnight against distilled water and lyophilized. cSPLUNC1 was suspended in 200 µl water and residual SDS was removed using a detergent removal column (Millipore, Billerica, MA). cSPLUNC1 was denatured by addition of 2 ml of suspension buffer (6 M guanidine-HCl, 20 mM Tris-HCl pH 7.0, 500 mM NaCl) and dialyzed at 4°C against buffers that contained a sequentially reduced amount of urea (6 M, 3 M, 1 M, 0.5 M, and 0.1 M) to promote the slow refolding of cSPLUNC1. Native cSPLUNC1 was ultimately dialyzed against water overnight, lyophilized, and suspended in water.

### Antimicrobial activity of native cSPLUNC1

The liquid bactericidal assay used was performed essentially as described [40]. The assay was conducted with increasing concentrations of native cSPLUNC1, the human cathelicidin AP LL-37 (Peprotech, Rocky Hill, NJ), or lysozyme (Fisher). Colony forming units were counted, and percent killing was calculated relative to identical cultures incubated with only sodium phosphate buffer (pH 7.2) that contained 1% sBHI. Data from triplicate assays were presented as mean percentage killed ± standard deviation relative to concentration of AP.

### Determination of the effect of a cSPLUNC1 knock down on NTHI survival in the chinchilla middle ear

NTHI is a predominant bacterial causative agent of OM that has been extensively utilized in chinchilla models [44]. As such, six chinchillas (two cohorts of three each) were used to assess the outcome of cSPLUNC1 knock down on the ability of NTHI to multiply and survive in the middle ear. Ten nanomoles of either a control or cSPLUNC1-specific siRNA were administered transbullarly, and all animals were challenged one day later with approximately 1000 cfu of NTHI strain 86-028NP [44]. Tympanometry was used to monitor for development and severity of OM as measured by: changes in middle ear pressure, tympanic width, and tympanic membrane compliance as described [45]. A tympanogram was considered abnormal if middle ear pressure was ≥−100 daPa or ≥+60 daPa, compliance values were ≤0.5 or ≥1.2 ml, and/or tympanic width was ≥150 daPa [33]. Epitympanic taps were attempted two and four days after bacterial challenge and collected fluids were maintained on ice until serially diluted and cultured on chocolate II agar plates (Fisher, Pittsburgh, PA). Bacterial counts were reported as the average cfu NTHI/ml middle ear fluid.

### Influence of a cSPLUNC1 knock down on morphology of middle ears challenged with NTHI

Micro-computed tomography (CT) (GE Healthcare, Pittsburgh, PA) imaging can be utilized to non-invasively monitor morphological changes within the middle ear cavity during development of OM. As such, we captured images of the chinchilla middle ear before administration of siRNA, one day after delivery of siRNA, and just prior to sacrifice. Final imaging was completed four days after bacterial challenge, which was five days after administration of siRNA to chinchillas. Anesthetized animals were scanned at 80 kV with a 450 µA current and a 3-D rendering from 200 separate images was generated with a reconstruction utility provided by the manufacturer. Images were exported as a tiff file and the tympanic membrane was pseudo-colored red for improved visualization.

Our micro-CT analysis of chinchillas administered cSPLUNC1 siRNA suggested that a reduction in cSPLUNC1 expression diminished the ability of the ET to function correctly during NTHI-induced OM. As such, we used a histological approach to compare the morphology of Eustachian tubes from animals that received control or cSPLUNC1 siRNA. Four days after bacterial challenge, bullae with the ET intact were removed and fixed in 2% paraformaldehyde overnight at 4°C. After fixation, samples were decalcified in a solution of 0.1 M tris (hydroxymethyl) aminomethane pH 6.95 and 0.35 M ethylenediaminetetraacetic acid (EDTA). EDTA solution was changed every 48 hours until no calcium was detected according to the method of Seilly *et al.* [46]. Once decalcified, samples were embedded in paraffin, 5 µm sections were cut and mounted onto slides, and sections were stained with hematoxylin and eosin. Images were captured with a Zeiss Axiocam MRc camera attached to a Zeiss Axioskop 40 microscope (Carl Zeiss Inc., Thornwood, NY).

### Surfactant activity of cSPLUNC1

The results that we obtained from our *in vivo* analysis suggested that cSPLUNC1 exhibited surfactant activity. We therefore tested the ability of purified native cSPLUNC1 to act as a surfactant *in vitro*. One measure of surfactant activity is the ability to reduce surface tension, thus promoting spread and reducing the contact angle of a solution on a hydrophobic surface. Four microliter droplets of water, 100 µg lysozyme/ml water (Fisher), 100 µg native cSPLUNC1/ml water, or SDS (10%) (Fisher) were thereby incubated on the hydrophobic surface of Parafilm M for one minute at room temperature as reported [36]. SDS was used as a positive control and lysozyme was used as a negative control as is not known to exhibit surfactant activity [36]. The concentration of cSPLUNC1 used in this analysis was based upon the estimate that cSPLUNC1 is present in epithelial airway secretions at concentrations of 10–250 µg/ml [28]. Samples were imaged with Brightfield optics on a Zeiss Stemi SV6 microscope. Diameter of a droplet was measured in millimeters using Axiovision software and values (n = 4) were standardized to the average diameter of a droplet of water and reported as the percentage increase in diameter relative to the water control. Statistical significance was determined using Student's T-test and a *p*-value ≤0.05 was considered significant.

We also used pendant drop tensiometry (Augustine Scientific, Newbury, Ohio) to directly measure surface tension of a 1 µl droplet of water, lysozyme, native cSPLUNC1, or SDS (10%). Several concentrations of cSPLUNC1 or lysozyme diluted in water (20 µg/ml, 100 µg/ml, and 500 µg/ml) were used in this procedure. In this analysis, a droplet of one of the four solutions was formed on the end of a capillary tip and digitally imaged. The mean curvature of the solution was determined at over 300 points along its surface, and surface tension was calculated from these values. Tension measurements were determined five times for each sample and mean values ± standard deviation were reported. Statistical significance was determined using Student's T-test and a *p*-value ≤0.05 was considered significant.

### Impact of silencing cSPLUNC1 on ET mucociliary clearance

Expression of surfactant proteins can promote effective mucociliary clearance in the airway [12]. We therefore determined if cSPLUNC1 expression and its surfactant activity (or lack thereof) affected mucociliary clearance in the URT. Chinchillas were transbullarly administered (n = 4 ears per cohort) saline, 10 nmoles of cSPLUNC1 siRNA or 10 nmoles of a negative control siRNA. Twenty-four hours later, the time required to transport a small bolus of 5% Coomassie brilliant blue (Fisher) solution, prepared in pyrogen-free saline, from the inferior aspect of the chinchilla middle ear cavity to the nasopharyngeal orifice of the ET was assessed following a published protocol [37]. The ability of chinchillas to transport dye from the middle ear to the nasopharyngeal orifice of the ET is a direct indicator of the mucociliary clearance capability of this tubal organ [37]. One cohort of animals that did not receive siRNA was administered 150 µl of a 0.005% solution of isoproterenol (Sigma), to increase ciliary beat frequency and mucociliary clearance, and thus served as a positive control [37]. One hundred and fifty microliters of dye was delivered transbullarly to all four cohorts of chinchillas and the transport time for appearance of dye at the nasopharyngeal orifice of the ET was measured in seconds. A camera (Watec-231S, Watec, Orangeburg, NY) equipped with a three inch otoscope (Sopro-Comeg, Tuttlingen, Germany) was used to visualize the opening of the ET and images were captured with computer software (VetDock 2.1, Med-Rx, Largo, Florida). Statistically significant differences in dye transport times between the cohort that received saline alone and those cohorts that received either isoproterenol, negative control siRNA, or cSPLUNC1-specific siRNA was calculated using Student's *t*-test with significance accepted at a *p*-value of ≤0.05.

## Supporting Information

Table S1 (0.06 MB DOC)

Materials and Methods S1 (0.06 MB DOC)

Figure S1 *In silico* analysis of cSPLUNC1. DNASTAR Clustal W alignment of cSPLUNC1 with human (accession no. AF70860) and rat SPLUNC1 (accession no. NM172031). Numbers to the left represent the amino acid number for the respective SPLUNC1 molecule and the arrow shows the putative signal sequence. Chinchilla SPLUNC1 was an ortholog of human SPLUNC1.
(7.14 MB TIF)

Figure S2 Growth of polarized chinchilla nasopharyngeal epithelial cells (CNPEs) and detection of native cSPLUNC1. Nasopharyngeal mucosa was recovered from a chinchilla and transferred to a Transwell membrane. When the culture was confluent, growth medium was removed from the apical surface to promote polarization of cells. (A) Hematoxylin and eosin stain of CNPEs cultured for 40 days at the air-liquid interface shows cellular stratification and differentiation. (B) Section of embedded CNPEs incubated with rabbit anti-cSPLUNC1 and goat anti-rabbit IgG-AlexaFluor 488 to detect cSPLUNC1. The section was also counterstained with the nuclear stain DAPI (blue). (C) Serial section from (B) stained with periodic acid-Schiff-Alcian blue to detect mucopolysaccharide-producing cells. Note that the cell producing cSPLUNC1 (yellow arrow in B) was identified as a mucin-producing goblet cell (black arrow in C) as indicated by the red color.
(0.76 MB TIF)
