# Supplementary material for: The Multifunctional Host Defense Peptide SPLUNC1 Is Critical for Homeostasis of the Mammalian Upper Airway
Source: PLoS One. 2010 Oct 7;5(10):e13224. doi: 10.1371/journal.pone.0013224 (PMC2951362; doi:10.1371/journal.pone.0013224)
Supplement: Table S1 — (0.06 MB DOC) [file pone.0013224.s001.doc]

**Table S1**. Primers, adapters, and plasmids used in this study.

| Name | Description | Source |
| --- | --- | --- |
| Primer |  |  |
| Biotin-*att*B2-Oligo(dT) | 5’-Biotin-ggcggccgcacaactttgtacaagaa a gttgggt(T)_19_-3’ | Invitrogen |
| *att*B1 adapter | 5’-tcgtcggggacaactttgtacaaaaaagtt gg-3’  3’-cccctgttgaaacatgttttttcaaccp-5’ | Invitrogen |
| Human SPLUNC1-F Internal | 5’-aagtgaatacgcccctggtc-3’ | P. B. McCray Jr. |
| Human SPLUNC1-R Internal | 5’-agaccttgacaaactgtagtcc-3’ | P. B. McCray Jr. |
| M13F | 5’-GTAAAACGACGGCCAG-3’ | Promega |
| M13R | 5’-CAGGAAACAGCTATGAC-3’ | Promega |
| Human SPLUNC1- F upstream | 5’-agaggagaccaggacagct-3’ | P. B. McCray Jr. |
| Chinchilla SPLUNC1-R internal  Chinchilla SPLUNC1-F upstream | 5’-AGACTTGTGATCATGGGC-3’  5’-ggctgagatcctgcgaaaccc-3’ | This study  This study |
| Human SPLUNC1-R downstream | 5’-cagcagaggccagccccttcc-3’ | This study |
| Chinchilla SPLUNC1 Overexpression-F | 5’-ggtggttgctcttccaaccagctcggtgg cctgccattgcc’-3’ | This study |
| Chinchilla SPLUNC1 Overexpression-R | 5’-ggtggttgctcttccgcactagaccttgat gacAAAttgtaccc-3’ | This study |
| Ssp DnaB intein forward | 5’-ACTGGGACTCCATCGTTTCT-3’ | This study |
| Mxe intein reverse | 5’- GGCACGATGTCGGCGATGC-3’ | This study |
| Chinchilla SPLUNC1-F | 5’-TTCCACTCTTGAACATCC-3’ | This study |
| Chinchilla SPLUNC1-R | 5’- AGACTTGTGATCATGGGC-3’ | This study |
| Chinchilla β-actin-F | 5’-AAGGACTCGTACGTGGGAGATGAAG-3’ | This study |
| Chinchilla β-actin-R | 5’-ATCTGGGTCATCTTCTCACGGTTAGC-3’ | This study |
| Human SPLUNC1 overexpression-F | 5’-GGTGGTTGCTCTTCCAACCAGTTTGGAGG CCT GCCCGTGCC-3’ | This study |
| Human SPLUNC1 overexpression-R | 5’-GGTGGTTGCTCTTCCGCATTAGACCTTGATG ACAAACTGTAGTCC -3’ | This study |
| Plasmid |  |  |
| pGMSH-9 | Partial cDNA of cSPLUNC1 cloned into pGEM-T  easy | This study |
| pGMSH-10 | Full-length cDNA of cSPLUNC1 cloned into  pGEM-T easy | This study |
| pTWIN-1 | Expression vector that encodes ampicillin resistance | New  England  Biolabs |
| pGM-15 | Expression vector that contains a chitin binding domain fused in-frame with an intein tag and the chinchilla SPLUNC1 coding region | This study |
| pGM-16 | Expression vector that contains a chitin binding domain fused in-frame with an intein tag and the human SPLUNC1 coding region | This study |
|  |  |  |
|  |  |  |
|  |  |  |
