# Supplementary material for: The Multifunctional Host Defense Peptide SPLUNC1 Is Critical for Homeostasis of the Mammalian Upper Airway
Source: PLoS One. 2010 Oct 7;5(10):e13224. doi: 10.1371/journal.pone.0013224 (PMC2951362; doi:10.1371/journal.pone.0013224)
Supplement: Materials and Methods S1 — (0.06 MB DOC) [file pone.0013224.s002.doc]

**Supplementary Material**

**Materials and Methods S1**

**Expression and purification of (r)cSPLUNC1.** Phusion DNA polymerase (New England Biolabs, Ipswich, MA), pGMSH-10, with chinchilla SPLUNC1 overexpression-F and chinchilla SPLUNC1 overexpression-R primers (Table S1) were used in a PCR reaction to amplify a 735-bp fragment that encoded the secreted cSPLUNC1 protein. Forward and reverse primers were designed to contain *Sap*I restriction sites and conditions for amplification were an initial denaturation at 98˚C followed by a thirty-five cycle 3-step procedure that consisted of denaturation at 98°C for 10 seconds followed by annealing at 62°C for 30 seconds and extension at 72°C for 30 seconds. PCR products were separated in an ethidium-bromide stained agarose gel and the Wizard SV gel and PCR clean-up system (Promega, Madison, WI) was used to extract and purify amplicons. The plasmid pTWIN-1 (New England Biolabs) and amplified DNA fragments were digested with *Sap*I, gel extracted again, and ligated (New England Biolabs). Plasmids obtained from this cloning step were transformed into *E. coli* Top10 cells for growth on LB supplemented with 50 μg ampicillin/ml. Plasmids from selected recombinants were sequenced (Table S1; Sap DnaB intein forward and Mxe intein reverse) and one (pGM-15) contained cSPLUNC1 in frame with the chitin binding domain and intein coding regions encoded by the plasmid vector.

The IMPACT system was used to purify (r)cSPLUNC1. In this approach, fusion protein is bound to a chitin resin and subsequent auto-cleavage of this fusion protein is induced when the temperature (with a shift from 4°C to room temperature) and pH (with a shift from an alkaline to more acidic pH) of the column are changed. These conditions allow for purification of a protein in a single step without the need to remove proteases which are typically used to separate affinity tags from recombinant molecules. We transformed pGM-15 into *E. coli* Rosetta (EMD chemicals, Gibbstown, NJ) and determined conditions necessary for expression of (r)cSPLUNC1. Three liters of cells in LB broth were grown to mid-log growth phase at 37°C and induced to express (r)cSPLUNC1 for four hours with 1 mM isopropylthio-β-galactoside. Bacterial cells were pelleted by centrifugation at 8000 X g for 10 minutes and the cell pellet was suspended in 20 ml of B-Per protein extraction reagent (Pierce, Rockford, IL) for 15 minutes on ice. Samples were sonicated three times for 20 seconds each and centrifuged at 15,000 X g for 30 minutes. (r)cSPLUNC1 was insoluble in B-Per and therefore this fraction was suspended in denaturation buffer (8 M urea, 20 mM Tris-HCl pH 8.5, 500 mM NaCl, and 1 mM EDTA) to solubilize protein. The preparation was centrifuged at 15,000 X g for 30 minutes and the supernatant was sequentially dialyzed at 4°C against buffers that contained a reduced amount (6 M, 3M, 1M, 0.5 M, and 0.1 M) of urea to allow the fusion protein to re-fold. The final product was applied to a chitin column (New England Biolabs) at 4°C. The fusion protein was allowed to bind to chitin for 1 hour at 4°C and any loosely bound protein was removed from the column with 80 ml of wash buffer (0.1 M urea, 20 mM Tris-HCl pH 8.5, 500 mM NaCl, and 1 mM EDTA). The column was further washed with 15 ml of elution buffer (0.1 M urea, 20 mM Tris-HCl pH 6.0, 500 mM NaCl, 1 mM EDTA) to provide a buffer exchange. The column (with the bottom plug in place) was then incubated with 5 ml of elution buffer and transferred from 4°C to room temperature to initiate intein-mediated auto-cleavage of the fusion protein. After 24 hours, the 5 ml elution buffer was collected, dialyzed against 50% ethanol, and lyophilized. Purified (r)cSPLUNC1 was suspended in 100 μl of 50% ethanol/50% PBS, pH 7.4.

**Expression and purification of (r) human SPLUNC1.**  Human SPLUNC1 was amplified from a construct in which human SPLUNC1 cDNA was cloned into a pBluescript II SK- vector (provided by P.B. McCray Jr.). Phusion DNA polymerase (New England Biolabs, Ipswich, MA) with human SPLUNC1 overexpression-F and human SPLUNC1 overexpression-R primers (Table S1) were used in a PCR reaction to amplify a fragment that encoded secreted SPLUNC1 protein. Cloning of the cDNA into pTWIN-1, protein overexpression, and subsequent purification of the recombinant protein was done as with (r)cSPLUNC-1.

**Growth of chinchilla nasopharyngeal epithelial cells *in vitro.*** To generate an abundant source of native cSPLUNC1, we cultured differentiated, pseudostratified, ciliated chinchilla nasopharyngeal epithelial cells (CNPEs). Upon sacrifice of anesthetized chinchillas, nasopharyngeal mucosa was dissected and placed in cell culture medium (741 ml DMEM [Mediatech, Manassas, VA], 247 ml Ham’s F-12 [Mediatech], 25 mM Hepes [Fisher], 200 mM glutamine [Mediatech], 0.4 ug/ml hydrocortisone (Sigma), 2 ug/ml Isoproterenol [Sigma], 10 ng/ml epidermal growth factor [Fisher], 5% fetal bovine serum [Fisher], 100 U Penicillin/Streptomycin [Mediatech], and 2.5 ug/ml Amphotericin B [Fisher] per liter). The explant was transferred to a sterile Petri dish and dissected into approximately 1mm^3^ fragments. Three pieces of tissue were placed on a 6-well Transwell insert (Fisher) that contained 2 ml of medium in the bottom chamber and 0.5 ml in the top chamber, and cells were incubated at 37°C with 10% CO_2_ for five days. Cells were incubated with growth medium supplemented with 10 ng epidermal growth factor (Fisher) / ml, and tissue explants were removed when cells were approximately 70% confluent. CNPEs were allowed to grow to confluence before medium was removed from the top chamber only, thereby allowing further culture of these cells at the air-fluid interface. While polarizing, cells were fed with CNPE growth medium via the lower chamber of the transwell every 2 days for 14 days. Selected transwell membranes on which CNPE cells had grown were embedded in paraffin, sectioned into 4-µm sections, and stained with hematoxylin and eosin to ensure polarization and cellular stratification.

**Immunofluorescent detection of cSPLUNC1 production by CNPEs.** We used immunofluorescent imaging and histology to begin to determine whether CNPEs expressed cSPLUNC1 and also what cell type produced the native protein. For immunofluorescent imaging, we incubated sections with image-iT FX signal enhancer (Invitrogen) to block non-specific binding, a 1:500 dilution of rabbit anti-cSPLUNC1, and a 1:200 dilution of goat anti-rabbit IgG-AlexaFluor 488 secondary antibody (Invitrogen) to detect cSPLUNC1. ProLong Gold antifade reagent (Invitrogen) that contained DAPI (4’,6’-diamino-2-pheynylindole) was used as a counterstain to label the nuclei of CNPEs. Sections were viewed using a Zeiss LSM 510 Meta confocal system attached to a Zeiss Axiovert 200 inverted microscope (Carl Zeiss Inc., Thornwood, NY).

In addition, periodic acid-Schiff-Alcian Blue staining was done according to standard methods to specifically label mucopolysaccharides and therefore identify mucous-producing goblet cells.

**Nano LC/MS/MS analysis of purified native cSPLUNC1**. To provide further evidence that we had indeed isolated native cSPLUNC1, electroeluted SPLUNC1 was subjected to SDS-PAGE, stained with Coomassie Brilliant blue (Fisher), and submitted for identification to the proteomics facility at The Ohio State University.

Trimmed gel pieces that contained SPLUNC1 (monomer and predicted multimer) were washed three times in nanopure water for 5 minutes and incubated for ten minutes with 1:1 (v/v) solution of methanol: 50 mM ammonium bicarbonate. The gel pieces were dehydrated with 1:1 (v/v) acetonitrile: 50 mM ammonium bicarobonate solution then incubated with 25 mM dithiothreitol in 100 mM ammonium bicarbonate. Gel bands were further incubated with 55 mM iodoacetamide in 100 mM ammonium bicarbonate for 30 minutes in the dark. The gel bands were washed again with two cycles of water and dehydrated with a 1:1 (v/v) acetonitrile: 50 mM ammonium bicarobonate solution. Protein in the gel slices were digested with 12 ng trypsin/ml in 0.01% ProteaseMAX Surfactant for 5 minutes and protease activity was stopped by addition of 0.5% trifluoroacetic acid. Capillary-liquid chromatography-nanospray tandem mass spectrometry (Nano-LC/MS/MS) was performed on a Thermo Finnigan LTQ mass spectrometer equipped with a nanospray source operated in positive ion mode. The LC apparatus was an UltiMate 3000 system (Dionex, Sunnyvale, CA). For separations, solvent A was water that contained 50 mM acetic acid and solvent B was acetonitrile. Five microliters of sample was first injected on a μ-precolumn cartridge (Dionex, Sunnyvale, CA), and washed with 50 mM acetic acid. A 5 cm x 75 μm ID ProteoPep II C18 column (New Objective, Inc. Woburn, MA) packed directly in the nanospray tip was used for chromatographic separations. Peptides were eluted directly from the column into the LTQ system using a gradient of 2-80% solvent B over 45 minutes, with a flow rate of 300 nl/min. The total run time was 65 minutes. The MS/MS was acquired according to standard conditions established in the proteomics facility. Briefly, a nanospray source operated with a spray voltage of 3 KV and a capillary temperature of 200°C was used. The sequence of the mass spectrometer was programmed for a full scan recorded between 350 – 2000 Da, and a MS/MS scan to generate product ion spectra was used to determine amino acid sequence in consecutive instrument scans of the ten most abundant peak in the spectrum. Dynamic exclusion was enabled with a repeat count of 2 within 10 seconds, a mass list size of 200, an exclusion duration 350 seconds, the low mass width was 0.5 and the high mass width was 1.5.

Sequence information from the MS/MS data was processed by converting data into a merged file using an in-house program, RAW2MZXML_n_MGF_batch. The resulting files were searched using Mascot Daemon (Matrix Science, Boston, MA) and the database searched against the full SwissProt database version 57.5 or NCBI database version 20091013. The mass accuracy of the precursor ions were set to 2.0 Da given that the data was acquired on an ion trap mass analyzer and the fragment mass accuracy was set to 0.5 Da. Two missed cleavages for the enzyme were permitted. A decoy database was searched to determine the false discovery rate (FDR) and peptides were filtered according to the FDR. Protein identifications were checked manually and proteins with a Mascot score of 50 or higher with a minimum of two unique peptides from one protein having a *-b* or *-y* ion sequence tag of five residues or better were accepted.

**Circular dichroism analysis of native SPLUNC1.** To provide evidence that isolated native SPLUNC1 folded correctly, circular dichroism (CD) analysis was performed (PrimeSyn lab Inc., Hillsborough, NJ) on a Jasco CD spectrophotometer J715 SN B019160524 equipped with a peltier temperature programmer (PTC-34WI).  Five hundred micrograms cSPLUNC1/ml water was transferred to a QS 1.0 mm cell and allowed to equilibrate for 5 minutes at 25°C.  Spectra data was collected from 200-260 nm for cSPLUNC1 and the vehicle control (water) with values reported representing the spectrum of cSPLUNC1 subtracted from this control. Jasco software 1.5308 was used to smooth the resulting spectrum and K2D2 software (<http://www.ogic.ca/projects/k2d2/k2d2_set1.pl>) was used to predict the protein secondary structure from the spectrum that was generated with the wavelength range set from 200 nm to 240 nm.

**Production of anti-native cSPLUNC1***.*  We immunized rabbits with three 10 μg doses of native SPLUNC1 to generate rabbit polyclonal antiserum (Spring Valley Laboratories, Sykesville, MD). Western blot was used to confirm that this antibody recognized the immunogen as well as recombinant cSPLUNC1.
