# Supplementary figures and images for: The Multifunctional Host Defense Peptide SPLUNC1 Is Critical for Homeostasis of the Mammalian Upper Airway
Source: PLoS One. 2010 Oct 7;5(10):e13224. doi: 10.1371/journal.pone.0013224 (PMC2951362; doi:10.1371/journal.pone.0013224)

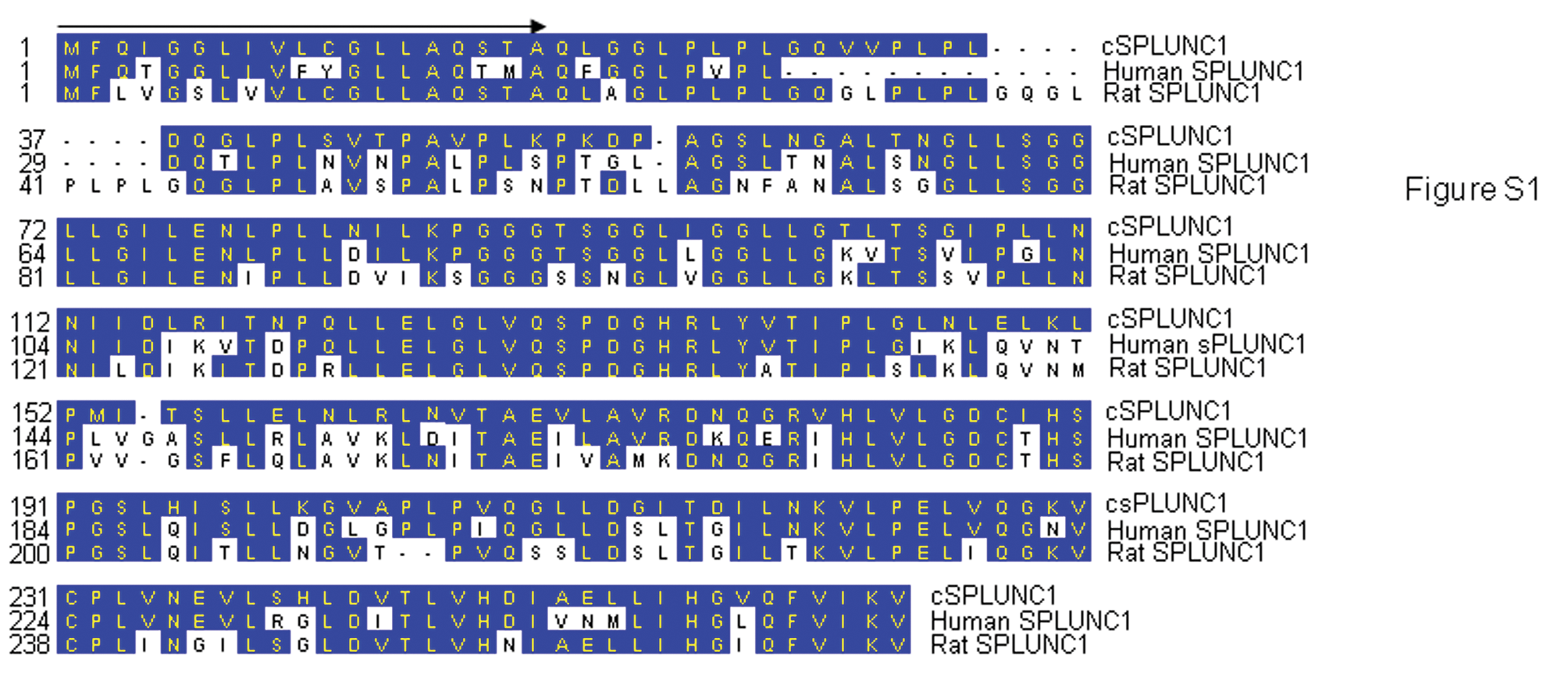

Supplement: Figure S1 — In silico analysis of cSPLUNC1. DNASTAR Clustal W alignment of cSPLUNC1 with human (accession no. AF70860) and rat SPLUNC1 (accession no. NM172031). Numbers to the left represent the amino acid number for the respective SPLUNC1 molecule and the arrow shows the putative signal sequence. Chinchilla SPLUNC1 was an ortholog of human SPLUNC1. (7.14 MB TIF) [file pone.0013224.s003.tif]

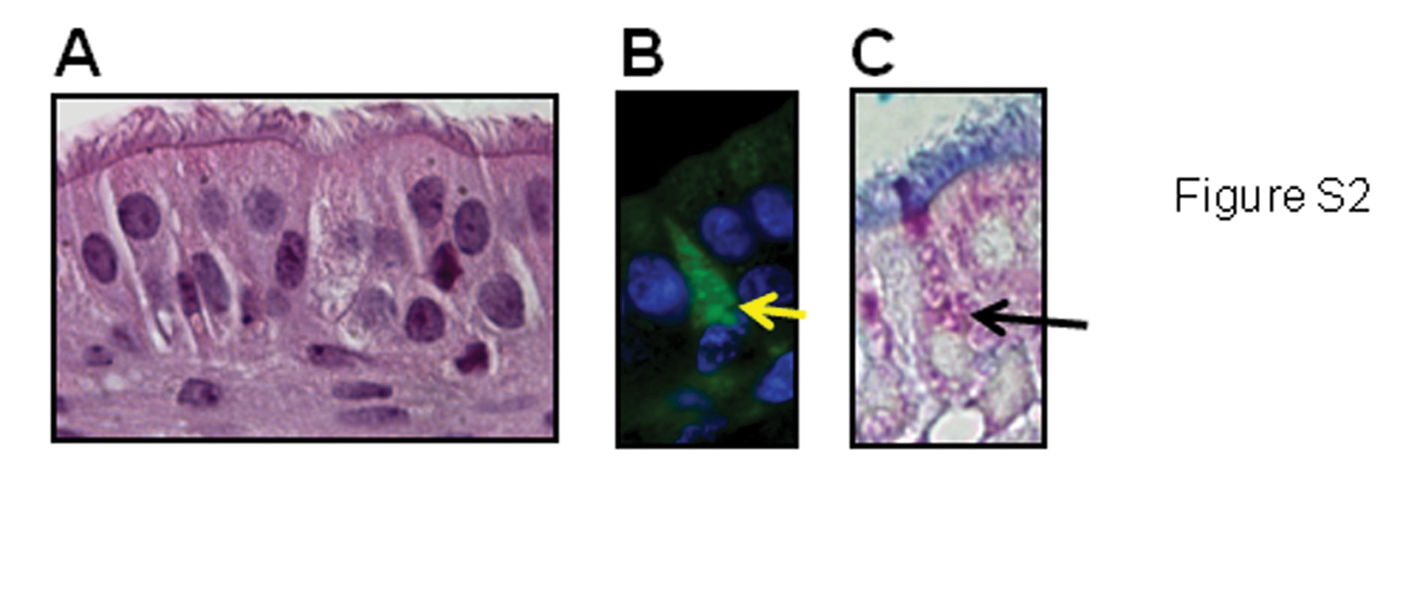

Supplement: Figure S2 — Growth of polarized chinchilla nasopharyngeal epithelial cells (CNPEs) and detection of native cSPLUNC1. Nasopharyngeal mucosa was recovered from a chinchilla and transferred to a Transwell membrane. When the culture was confluent, growth medium was removed from the apical surface to promote polarization of cells. (A) Hematoxylin and eosin stain of CNPEs cultured for 40 days at the air-liquid interface shows cellular stratification and differentiation. (B) Section of embedded CNPEs incubated with rabbit anti-cSPLUNC1 and goat anti-rabbit IgG-AlexaFluor 488 to detect cSPLUNC1. The section was also counterstained with the nuclear stain DAPI (blue). (C) Serial section from (B) stained with periodic acid-Schiff-Alcian blue to detect mucopolysaccharide-producing cells. Note that the cell producing cSPLUNC1 (yellow arrow in B) was identified as a mucin-producing goblet cell (black arrow in C) as indicated by the red color. (0.76 MB TIF) [file pone.0013224.s004.tif]
